# Nested Neuronal Dynamics Orchestrate a Behavioral Hierarchy across Timescales

**DOI:** 10.1016/j.neuron.2019.10.037

**Published:** 2020-02-05

**Authors:** Harris S. Kaplan, Oriana Salazar Thula, Niklas Khoss, Manuel Zimmer

**Affiliations:** 1 Department of Neurobiology, University of Vienna, Althanstrasse 14, 1090 Vienna, Austria; 2 Research Institute of Molecular Pathology (IMP), Vienna BioCenter (VBC), Campus-Vienna-BioCenter 1, 1030 Vienna, Austria

**Keywords:** neuronal dynamics, behavioral hierarchy, quantitative behavior, behavior organization, whole-brain imaging, motor control, ethology, *C. elegans* neuroscience, neuronal oscillations, hierarchical organization

## Abstract

Classical and modern ethological studies suggest that animal behavior is organized hierarchically across timescales, such that longer-timescale behaviors are composed of specific shorter-timescale actions. Despite progress relating neuronal dynamics to single-timescale behavior, it remains unclear how different timescale dynamics interact to give rise to such higher-order behavioral organization. Here, we show, in the nematode *Caenorhabditis elegans*, that a behavioral hierarchy spanning three timescales is implemented by nested neuronal dynamics. At the uppermost hierarchical level, slow neuronal population dynamics spanning brain and motor periphery control two faster motor neuron oscillations, toggling them between different activity states and functional roles. At lower hierarchical levels, these faster oscillations are further nested in a manner that enables flexible behavioral control in an otherwise rigid hierarchical framework. Our findings establish nested neuronal activity patterns as a repeated dynamical motif of the *C. elegans* nervous system, which together implement a controllable hierarchical organization of behavior.

## Highlights

• Slow dynamics across brain and motor circuits drive upper-hierarchy motor states
• Fast dynamics in motor circuits drive lower-hierarchy movements within these states
• Slower dynamics tightly constrain the state and function of faster ones
• This rigid hierarchy nevertheless enables flexible behavioral control

## Graphical Abstract

**Figure:**
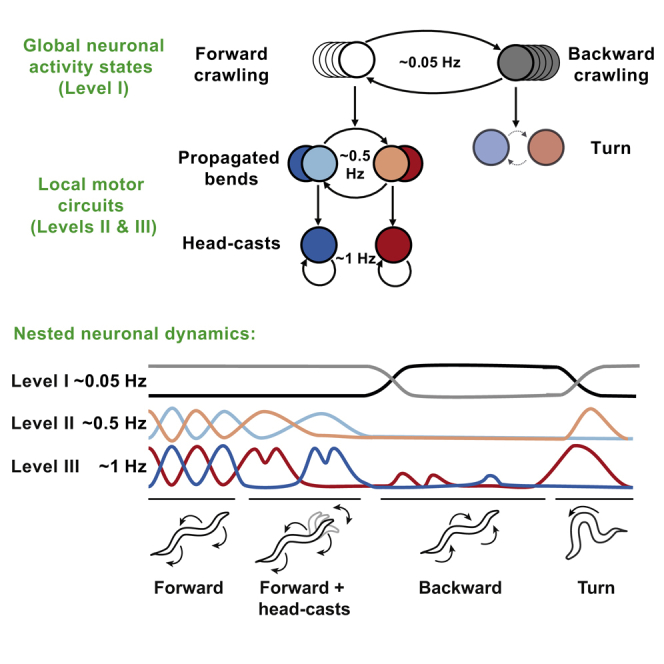

## Teaser

The nervous system must coordinate behavior across several timescales. A decades-old hypothesis in ethology posits that this occurs via hierarchical control. Kaplan et al. show that *C. elegans* employs hierarchically nested neuronal dynamics across brain and motor circuits to implement such multi-timescale behavioral organization.

## Introduction

Animal behavior unfolds over a wide range of timescales, from sub-second muscle contractions to circadian rhythms. Nervous systems not only act on each of these timescales but must also coordinate across them to implement long-term behavioral strategies and avoid interfering actions. Classical (Dawkins, 1976, Tinbergen, 1951) and modern (Berman et al., 2016, Glaze and Troyer, 2006, Gomez-Marin et al., 2016, Marques et al., 2018, Wiltschko et al., 2015; Duistermars et al., 2018) ethological studies posit that such inter-timescale coordination is hierarchical, with longer timescales at higher hierarchical levels. Specific shorter-timescale actions are constrained to occur only in the context of a hierarch, a particular longer-timescale motor program or behavioral state (Dawkins, 1976). Tinbergen described stickleback behavior as hierarchical, with the reproductive instinct encompassing sub-behaviors like nest building or defensive fighting; each of these, in turn, consists of specific sub-actions like digging or biting (Tinbergen, 1951). Modern quantitative analyses suggest that behaviors as diverse as birdsong and *Drosophila* locomotion are organized hierarchically (Berman et al., 2016, Glaze and Troyer, 2006).

While behavioral evidence supports the hierarchical model, neurophysiological evidence is lacking. It thus remains possible that the underlying neuronal mechanisms are entirely non-hierarchical (Berman et al., 2016). For example, while birdsong can be described hierarchically (Glaze and Troyer, 2006), neurophysiological evidence suggests a sequential chain of neuronal activities as the underlying mechanism (Long et al., 2010). In rodents, distinct behavioral timescales are represented by separable neuronal populations (Jin and Costa, 2015, Moore et al., 2013), which potentially provide a substrate for inter-timescale coordination. Rhythmic orofacial behaviors operating at different frequencies appear to be coordinated by a hierarchical phase-resetting mechanism, according to behavioral and anatomical evidence (Moore et al., 2013). Still, it remains unclear how different timescale neuronal dynamics interact to implement hierarchical behavior.

Addressing this question necessitates fine-grained analysis of both behavior and neuronal activity (Krakauer et al., 2017). We therefore examined *C. elegans* locomotion. *C. elegans* behaviors include sub-second body-bends, seconds- to minutes-long motor states such as forward and reverse crawling, and longer-lasting states of exploration, exploitation, and quiescence. Detailed analyses of postural dynamics have hinted at an underlying hierarchical organization (Gomez-Marin et al., 2016). Physiological studies, however, have primarily focused on single timescales. For example, calcium imaging studies have described motor neuron dynamics underlying the oscillatory gait (Gao et al., 2018, Shen et al., 2016, Wen et al., 2012, Xu et al., 2018, Fouad et al., 2018) and interneuron dynamics underlying forward/reverse locomotion switches (Kato et al., 2015, Kawano et al., 2011, Li et al., 2014, Piggott et al., 2011).

Here, we use quantitative behavior analysis, nervous-system-wide single-cell-resolution functional imaging, and circuit manipulations to reveal nested neuronal dynamics giving rise to a behavioral hierarchy in *C. elegans*. At the uppermost hierarchical level, an ∼0.05-Hz nervous-system-wide neuronal activity cycle drives switches between forward and reverse crawling states and also controls two faster neuronal oscillations, toggling them between different dynamical states and functional roles. At the intermediate hierarchical level, B-class motor neurons (B-MNs) drive ∼0.5-Hz crawling undulations. At the lowest hierarchical level, SMD motor neurons drive ∼1-Hz head-casts, which are further nested within specific phases of the mid-level oscillation. Crucially, by comparing neuronal activity in moving versus immobilized animals, we show that these nested dynamics persist in the absence of behavioral execution. Hierarchically nested neuronal dynamics are therefore an intrinsic property of these neurons and their circuit interactions.

## Results

### A Multi-timescale Behavioral Hierarchy of Motor Actions

**Figure 1:**
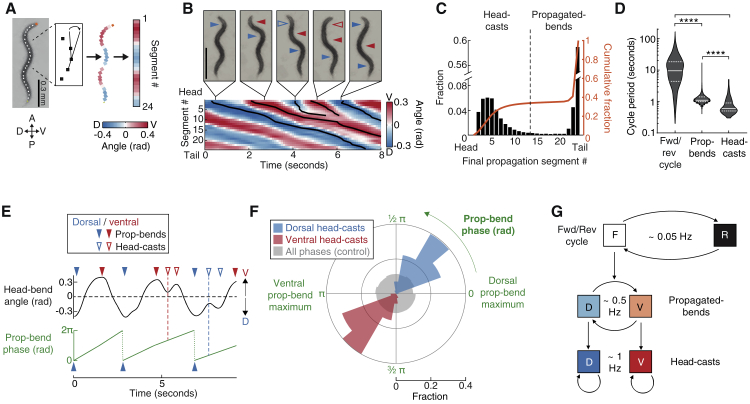
A Multi-timescale Behavioral Hierarchy (A) Body angle measurement. A, anterior; P, posterior; V, ventral; D, dorsal. (B) Lower: example posture time series kymogram. Head-bend propagations traced by black lines. Upper: worm images with propagated-bends (filled arrows) and head-casts (open arrows) indicated. Scale bar, 0.4 mm. (C) Fractional and cumulative distributions of each head bend’s most posterior propagation segment. Dotted line indicates cutoff used to distinguish head casts and propagated bends in subsequent quantifications. n = 45,129 head bends pooled from 28 assays, ∼20 animals per assay. (D) Violin plot showing median and 1^st^ and 3^rd^ quartiles of cycle periods for forward/reverse (forward + reverse bout duration, n = 1,259), propagated-bend (n = 25,817), and head-cast cycles (n = 341) pooled from 14 assays, ∼20 animals per assay. ^∗∗∗∗^p < 0.0001, Mann-Whitney test. (E) Example head-bend angle time series illustrating propagated-bend phase measurement. Red and blue dashed lines indicate initial head-cast phases, quantified in (F). (F) Fractional distributions of initial head-casts binned according to their propagated-bend oscillation phases. n = 427 dorsal and 194 ventral head-casts pooled from 21 animals. p < 10^−6^ for both distributions, indicating the probability that each is drawn from the full data distribution shown in gray. (G) Hierarchical model of behavior with approximate cycle frequencies. See also Figure S1.

To identify behaviors with relationships across timescales, we video-tracked animals to measure motor programs and associated gaits. *C. elegans* switch stochastically between forward- or reverse-directed crawling (Roberts et al., 2016); each of these motor programs consists of alternating dorsal/ventral undulatory bends. During the predominant forward locomotion state, bends originate in the head and propagate posteriorly. We quantified 24 bend angles along the worm’s body (Figure 1A) and traced each head bend’s propagation to its posterior-most segment (Figures 1B and S1A). This revealed a bimodal distribution, indicating two distinct types: one propagating completely to the tail (henceforth “propagated-bends”) and another terminating anterior to the mid-body (henceforth “head-casts”) (Figures 1B and 1C). Several metrics suggested that head-casts were a distinct class of actions, as opposed to aborted propagated-bends (Figures S1B–S1D). Head-casts were reminiscent of previously described “foraging” movements of the nose (Huang et al., 2008). The exact definition of foraging is ambiguous, and previous measures likely included head-casts as well as faster, more anterior nose movements (for discussion, see Yemini et al., 2013). Here, we rely on a precise definition based on propagation and, therefore, use “head-casts” instead of “foraging.” Forward locomotion consisted primarily of propagated-bends (Figure 1C) intermittently superimposed with episodes of head-cast oscillations (Figures S1A and S1E); when they did occur, head-cast oscillation cycles were typically faster than propagated-bend ones (Figure 1D). Together with forward/reverse locomotion switches, these behaviors occurred on three distinct timescales (Figure 1D).

Nevertheless, these behaviors were considerably coupled. Head casts were ventral- or dorsal-biased, depending on whether the previous propagated-bend was ventral or dorsal (Figures 1E, upper, and S1F). To investigate this further, we calculated the phase of the propagated-bend cycle, with dorsal – ventral – dorsal corresponding to 0 – 1π – 2π rad (Figure 1E, lower). This revealed that head-cast episodes were only initiated at restricted phases (Figure 1F). This phase dependence, which we term “phase-nesting,” suggests a brief window of opportunity for head-casts within each propagated-bend cycle. Indeed, head-casts preferentially occurred during the longest-duration propagated-bend cycles, consistent with longer cycles having wider windows of opportunity (Figures S1G–S1I). Alternatively, head-cast occurrence could stall and, therefore, lengthen the propagated-bend cycle, a possibility we examine below. Both head-bend types occurred exclusively during forward locomotion: during reversals, bends propagated from tail to head, and no head-casts occurred (Figures S1J and S1K). Together, these data suggest a hierarchical organization of behavior, in which shorter-timescale behaviors are strictly constrained by specific longer-timescale behavioral phases or states (Figure 1G). We next examined neuronal activities underlying these behaviors.

### A Nervous-System-wide Representation of the Uppermost Hierarchical Level

In our previous work, we developed a single-cell-resolution, whole-brain, Ca^2+^-imaging approach in immobilized animals (Kato et al., 2015, Schrödel et al., 2013). Using principal-component analysis (PCA), we reported a low-dimensional, brain-wide activity cycle that dominates head ganglia. This included descending (and other) interneurons, which, in freely moving animals, were strictly active during forward or reverse locomotion (Kato et al., 2015), the uppermost level of our hierarchical model. These same neurons show coordinated dynamics during immobilization, with relationships to each other as expected from their behavior correlations (i.e., forward-active neurons were positively correlated with each other and negatively correlated with reverse-active neurons). Therefore, specific neuronal activity patterns in immobilized worms can be ascribed to forward or reverse motor command states (Kato et al., 2015).

**Figure 2:**
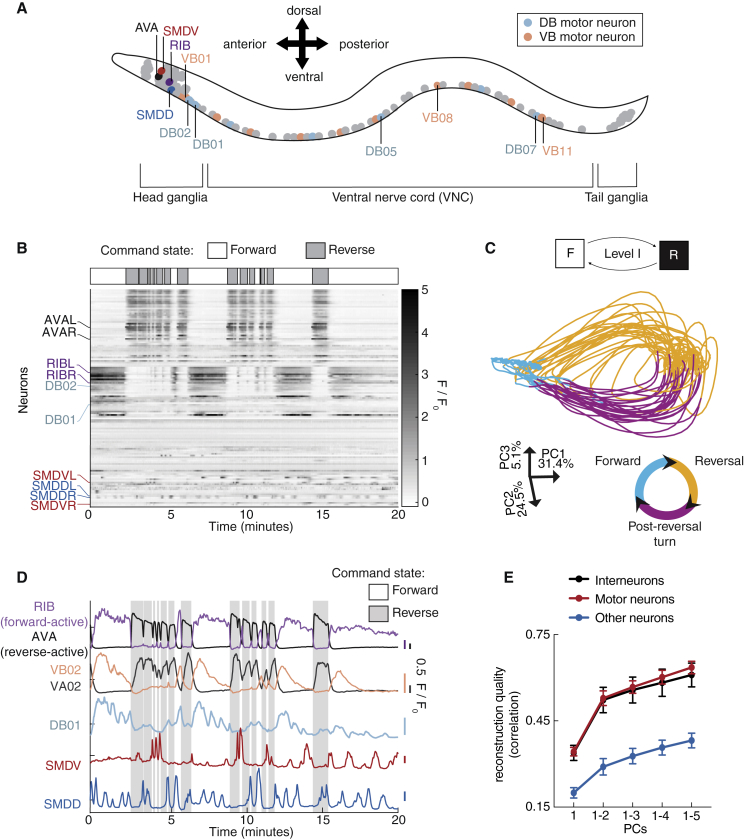
A Nervous-System-wide Representation of the Uppermost Hierarchical Level (A) Worm schematic. Neurons investigated in subsequent panels are labeled. (B–D) Example whole-nervous-system GCaMP6f recording. (B) Upper: motor command states inferred from neuronal activity (STAR Methods). Lower: fluorescence time series of 129 neurons, sorted by correlation. (C) Low-dimensional representation of nervous-system-wide activity, from principal-component analysis (PCA). Color key indicates motor command state inferred from neuronal activity (STAR Methods; arrowheads indicate directional flow). Coordinates depict principal component (PC) axes orientations and % variance explained. (D) Activity traces of selected neurons. Scale bars on the right represent 0.5 ΔF/F_0_, colored according to corresponding trace. (E) Mean ± SEM correlation coefficient between neuronal activity traces and traces reconstructed using indicated top PCs. n = 5 datasets. See also Figure S2 and Video S1.

Here, we expanded this approach beyond head ganglia to the entire nervous system, including ventral nerve cord (VNC, containing body motor neurons) and tail ganglia (n = 5; Figures 2A–2D and S2A; Video S1). Using PCA, we found low-dimensional dynamics strikingly similar to those restricted to head ganglia (Figures 2C and S2B). Notably, interneuron and motor neuron activities were equally well represented in this low-dimensional PCA space (Figure 2E), and both neuron classes showed strong modulations by forward/reverse command state (Figure S2C). Network activity corresponding to motor commands therefore extends from head ganglia to the motor periphery (Figure 2D), consistent with previous work showing pre-motor interneuron control of VNC motor neurons (Kawano et al., 2011, Xu et al., 2018). The AVB (Xu et al., 2018) and AVA (Kawano et al., 2011) pre-motor interneurons have been shown to exert powerful control over VNC activity and are likely responsible for connecting interneuron and motor neuron population dynamics into one coordinated signal. Accordingly, our results show that this signal permeates many VNC neurons even in completely immobilized animals (Figures 2E and S2C). In conclusion, a long-timescale, nervous-system-wide neuronal activity cycle underlies the uppermost hierarchical level.

Video S1. Whole-Nervous-System Ca^2+^ Imaging, Related to Figure 2 Maximum intensity projection of an example 30-min whole-nervous-system Ca^2+^ imaging recording, 60x real-time. Worm orientation and curvature as in Figure S2A: upper right neurons are in head ganglia, lower right are tail ganglia, and others are VNC.

### Identification of Candidate CPG Circuits for Lower Hierarchical Levels

**Figure 3:**
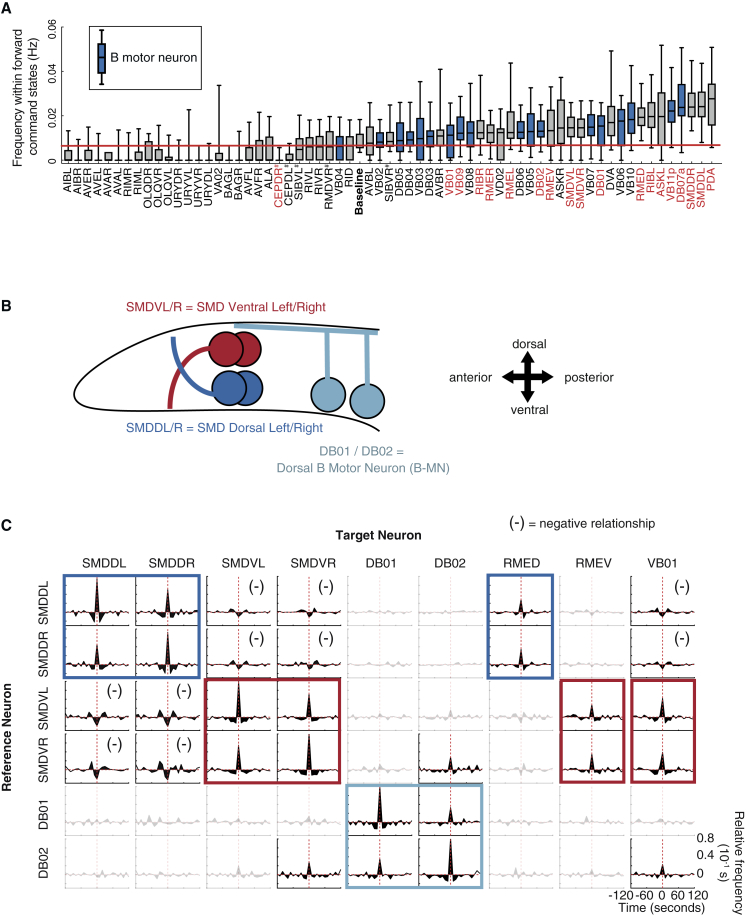
Identification of Candidate CPG Circuits for Lower Hierarchical Levels (A) Activity peak frequencies within each forward command state for all identified neurons, pooled from n = 5 whole-nervous-system and n = 5 head ganglia recordings. Median, interquartile, and 5%–95% range shown. “Baseline” distribution for one peak per forward command state; red line indicates its median. Neurons above “baseline” peaked multiple times within forward command states. Red labels indicate significantly non-random inter-peak interval distributions (STAR Methods). See Table S2 for multiple-comparison corrected p values and n numbers (number of forward command states = number of data points). ^#^ambiguous IDs, see STAR Methods for alternatives. (B) Worm head schematic illustrating positions and lateralized muscle innervations of CPG candidate motor neurons (not to scale). (C) Covariograms of SMD and DB with all coordinated neurons. All identified neurons were tested, but only those with significant correlation with at least one reference neuron are shown. Plots show relative frequencies of Ca^2+^ peaks of neurons in columns triggered to those in rows. Positive/negative values show higher/lower correlation than estimated by chance. Opaque plots differ significantly from random surrogate distributions (p values in Table S3). Colored borders denote significant positive relationships consistent within neuron classes. “(–)” denotes negative relationships. See also Figures S2 and S3.

PCA is sensitive to global correlations in time series data but less so to local and transient neuronal activity fluctuations. Therefore, we used additional approaches to screen our datasets for shorter-timescale activities underlying lower hierarchical levels. Rhythmic behaviors such as head bending are typically driven by central pattern generators (CPGs), neurons or circuits that can generate motor-pattern-like activity without any external feedback (Kiehn, 2011, Marder and Bucher, 2001). As our immobilized worm recordings lack dynamic proprioceptive inputs, they are particularly well suited for identifying CPG candidates. Behavioral studies have implicated B-MNs, along with an unknown head neuron class, as potential CPGs for forward locomotion (Fouad et al., 2018, Xu et al., 2018). Eighteen B-MNs distributed along the worm’s anterior-posterior axis synapse locally onto either dorsal (for DB01-DB07 B-MNs) or ventral (for VB01-VB11 B-MNs) muscle and are crucial for forward locomotion (Chalfie et al., 1985) (Figure 2A). Based on anatomical features (STAR Methods), we were able to identify most individual B-MNs in our data. We indeed found shorter-timescale fluctuations: some neuron classes, including certain B-MNs, showed several Ca^2+^ peaks nested within single forward command states (Figure 3A). Most of these neurons rarely peaked during reverse command states (Figure S2D). We focused on rhythmically active neuron classes, which we define as those with non-random inter-peak intervals (Figure 3A; STAR Methods). Two of these were excitatory motor neuron classes targeting head and neck muscle, making them top CPG candidates: SMD neurons (dorsal-projecting SMDDL and SMDDR and ventral-projecting SMDVL and SMDVR) and B-MNs DB01 and DB02 (Figures 2D and 3B). We hereafter refer to these neuronal classes as “oscillators,” given their rhythmic activity (Figures 3A and S3E–S3H).

To determine whether SMD and DB01/02 were part of larger oscillator circuits, we performed an appropriate variant of cross-correlation analysis: we calculated covariograms, which are shuffle-corrected, peri-event time histograms of one neuron’s Ca^2+^ peaks relative to another (Brody, 1999) (Figure 3C). This analysis delineated three small oscillator circuits (highlighted in different colors in Figure 3C): (1) SMDD and GABAergic ventral-projecting RMED head motor neurons oscillate in synchrony; (2) SMDV, dorsal-projecting RMEV, and the VB01 B-MN oscillate in synchrony and antagonistically with the SMDD oscillator (also see Hendricks et al., 2012, Shen et al., 2016); and (3) DB01 and DB02 oscillate in synchrony with weak correlations with the SMDV oscillator.

CPGs typically show both rhythm-generating (regularly repeating) and pattern-generating (coordinated across neurons, e.g., those controlling antagonistic muscles) activity (discussed in Kiehn, 2011). SMDD/V oscillator antagonism is consistent with both rhythm and pattern generation for antagonistic dorsal/ventral head bending. In contrast, DB01/02 oscillations showed positive correlations with VB01 activity (Figure 3C), despite targeting opposing muscles. Other B-MNs also exhibited rhythmic activity (Figure 3A), but we found no significant coordination among them (Figure S2E). These data are consistent with previous studies implicating B-MNs as distributed rhythm generators (Fouad et al., 2018), and they further suggest that proprioceptive feedback (described for B-MNs in Wen et al., 2012, Xu et al., 2018) is required for B-MN pattern generation. SMDs were also reported to be proprioceptive (Yeon et al., 2018), presumably in addition to the activity we report in immobilized animals. We suggest that proprioception is required for both SMD and B-MN frequency entrainment, as we observed a ∼10-fold reduction of frequency upon immobilization (compare Figure 3A with head-bend frequencies in moving animals, shown in 1D); such a reduction is consistent with CPG studies in other species (Fox et al., 2006, Goulding, 2009). For the SMDs, we confirmed that our measured activity frequencies were not limited by our acquisition rate, using a separate set of single-plane 50-Hz recordings (Figure S3). For other neurons, we cannot exclude the possibility of faster dynamics not captured by our 1–3 Hz volumetric recordings. In summary, in addition to slow global dynamics, immobilized animal recordings also revealed faster local dynamics, with the head motor neurons SMD and DB01/02 exhibiting largely independent rhythmic activity.

### SMDs and VNC Neurons Are Required for Head-Casts and Propagated-Bends, Respectively

**Figure 4:**
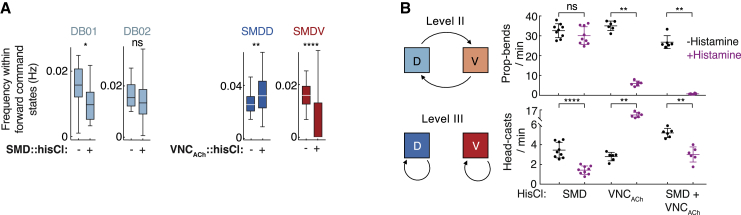
SMDs and VNC Neurons Are Required for Head-Casts and Propagated-Bends, Respectively (A) Frequencies as in Figure 3A for the indicated neurons in control, VNC_ACh_-inhibited, and SMD-inhibited animals, pooled across paired neurons, and across n = 5 (control, SMD::HisCl) or 4 (VNC_ACh_::HisCl) recordings. ^∗^p < 0.05, ^∗∗^p < 0.01, ^∗∗∗∗^p < 0.0001, Mann-Whitney test. ns, not significant. Boxplots show median, interquartile, and range. (B) Effects of SMD (n = 9), VNC_ACh_ (n = 6), and SMD+VNC_ACh_ (n = 6) inhibition on propagated-bends (upper) and head-casts (lower), mean ± SD. Each data point is the mean of an experimental repeat with ∼20 animals each. ^∗∗^p < 0.01, ^∗∗∗∗^p < 0.0001, Mann-Whitney test. ns, not significant. See also Figure S4.

To interrogate SMD and DB function, we transiently inhibited each using the histamine-gated chloride channel hisCl (Pokala et al., 2014). We first sought to confirm the oscillators’ independence in immobilized worms. Whole-brain recordings revealed DB01/02 oscillations in the absence of SMD activity (albeit with reduced DB01 frequency; Figure 4A). To examine SMD activity in DB01/02-inhibited animals, we generated VNC_ACh_::hisCl animals, in which all B-MNs, including DB01/02, are inhibited, among other cholinergic VNC neurons (no specific DB01/02 genetic driver is available). Whole-brain imaging in VNC_ACh_::hisCl animals showed increased SMDD and decreased SMDV oscillation frequencies (Figure 4A). This differential effect could be explained by inhibition of VB01, which is the only B-MN showing activity tightly coupled to SMD activity (positively with SMDV and negatively with SMDD; Figure 3C) and the only B-MN synaptically connected to the SMDs (a gap junction to SMDVR). Regardless of the SMDD/V balance, these data show that SMD and DB01/02 can operate as independent oscillators.

We next examined how oscillator inhibition affects locomotion. SMD::hisCl animals exhibited normal propagated-bend frequency but largely reduced head-cast frequency (Figures 4B, S4A, and S4B). VNC_ACh_::hisCl animals exhibited reduced propagated-bend frequency and increased head-cast frequency (Figures 4B, S4A, and S4B). Combining VNC_ACh_::hisCl and SMD::hisCl rendered animals almost completely immobile, with abnormally non-rhythmic head-cast-like bends (Figures 4B and S4A–S4E). In these animals, 11 head motor neuron classes were not targeted via hisCl but could not compensate for SMD and VNC_ACh_ inhibition, suggesting an especially prominent role for SMDs and VNC_ACh_ neurons. Altogether, these data suggest that the SMD and B-MNs are independently required for head-casts and propagated-bends, respectively.

These behavioral experiments also provided support for two predictions of our hierarchy model. First, reduced head-cast frequency in SMD::hisCl animals did not result in shorter propagated-bend cycle periods (Figures S4C and S4D), suggesting that head-cast occurrence does not stall or lengthen the propagated-bend cycle. Second, VNC_ACh_::hisCl showed a reciprocal effect on propagated-bends and head-casts (Figure 4B), consistent with longer propagated-bend cycles (Figure S4D) permitting wider windows of opportunity for head-casts. By contrast, reduced head casting in SMD::hisCl animals did not affect propagated-bend frequency (Figure 4B), consistent with a top-down effect of the longer-timescale behavior on the shorter-timescale one but not vice versa.

### Hierarchy Level I and II Interaction: SMD Neurons Are Multi-functional

**Figure 5:**
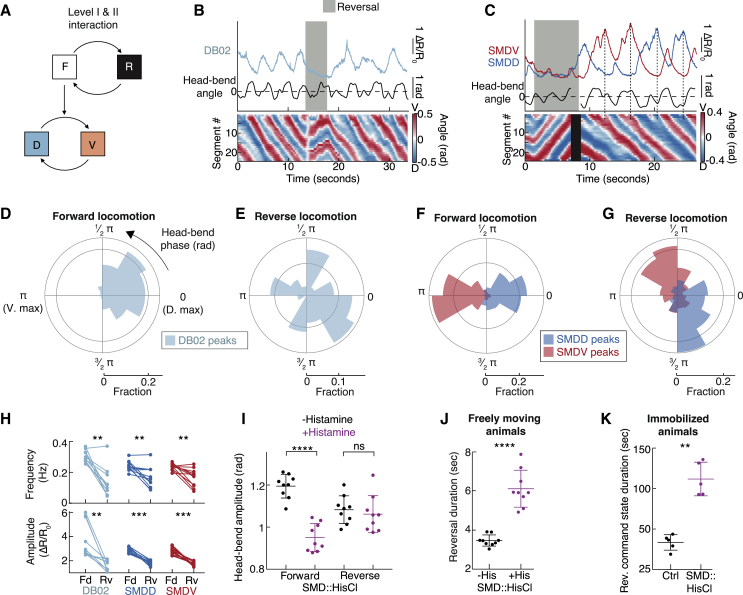
Hierarchy Level I and II Interaction: SMD Neurons Are Multi-functional (A) Hierarchy levels investigated. (B and C) Example DB02 (B) and SMD (C) Ca^2+^-imaging in moving animals. ΔR/R_0_ = normalized GCaMP/mCherry ratio. Lower: posture kymograms, black indicates missing data. Dotted vertical lines: unilateral SMDD- or SMDV-only oscillations. (D–G) Fractional distributions of DB02 (D and E) or SMD (F and G) Ca^2+^ peaks binned by head-bend phase during forward (D and F) or reverse (E and G) locomotion. n = 746 (D), 35 (E), 478 (F, SMDD), 389 (F, SMDV), 150 (G, SMDD), and 158 (G, SMDV) peaks pooled across 11 (SMD) and 10 (DB02) animals. p < 10^−6^ for all distributions except SMDV (p = 3 × 10^−6^) and DB02 (ns, p = 0.26) during reverse locomotion, indicating the probability that distributions are drawn randomly. Probability that each neuron’s reversal distribution was drawn from its respective forward distributions: p < 10^−6^ (SMDD and SMDV) and 0.0014 (DB02). (H) Frequency (upper) and average amplitude (lower) of detected activity peaks. ^∗∗^p < 0.01, ^∗∗∗^p ≤ 0.001, Wilcoxon matched-pairs signed rank test. n = 11 (SMD) and n = 10 (DB02) animals. (I) Peak head-bend amplitudes in SMD::hisCl, mean ± SD. ^∗∗∗∗^p < 0.0001, Mann-Whitney test; ns, not significant. (J) Reversal duration in SMD::hisCl, mean ± SD. ^∗∗∗∗^p < 0.0001, Mann-Whitney test. Each data point in (I) and (J) is the mean of an experimental repeat (n = 9) with ∼20 animals each. (K) Reversal command duration from immobilized head ganglia imaging, mean ± SD. Each data point is the mean from one animal, n = 5 each condition. ^∗∗^p < 0.01, Mann-Whitney test. See also Figure S5 and Video S2.

To determine how these circuits drive hierarchical behaviors, we performed Ca^2+^ imaging of SMDs and DB02 in freely moving animals (Figures 5B and 5C; Video S2). Fourier analysis revealed peaks in neuronal activity power spectra overlapping with peaks in head-bend angle power spectra, as expected for motor neurons targeting head muscle (Figures S5A–S5C). These data also confirmed that SMD neurons’ activities fluctuated about an order of magnitude faster in moving versus immobilized animals (compare Figures S5A and S5B to S3E–S3H). We next asked how the uppermost hierarchical level impacts faster-timescale activities (Figure 5A). As both SMDs and DB02 target head muscle, we examined neuronal activity relative to the head-bend oscillation (corresponding to hierarchy level II). During forward locomotion, SMDD and DB02 peaked during dorsal bends, SMDV peaked during ventral bends, and each neuron peaked during nearly every head-bend cycle (Figures 5D, 5F, and S5D). During reverse locomotion, DB02 and SMD activities were altered in different ways. DB02 activity was largely reduced, showing small peaks with no correlation to head bending (Figures 5B, 5E, 5H, and S5D). SMDs peaked at altered head-bend phases (Figure 5G) with less reliability (Figure S5D) and with lower frequency and amplitude (Figure 5H) compared to forward states. These altered SMD activity patterns suggest altered SMD function during reversals. Consistent with this idea, SMD inhibition caused reduced head-bend amplitude only during forward locomotion (Figure 5I).

Video S2. SMD Imaging in a Freely Moving Animal, Related to Figure 5 An example recording of neuronal activity and behavior, same as Figure 5C. Left: same animal with neural activity imaged at 63× (upper) and behavior at 4× (lower, segments for angle measurements marked). Right: neuronal activity (upper) and posture kymogram (lower).

If SMD activity during reversals does not promote head bending, what function does it serve? We reliably observed SMD Ca^2+^ peaks at reverse-to-forward transitions (Figure S5E). These transitions are typically accompanied by reorientation turns (Donnelly et al., 2013, Gray et al., 2005); the amplitude of these post-reversal turns correlated with SMD Ca^2+^-peak amplitude (Figure S5F), with mutually exclusive SMDV activity during ventral turns and SMDD activity during dorsal turns (Figure S5E). We therefore hypothesized that SMD reversal activity increases reverse-to-forward transition probability and promotes post-reversal turn amplitude. Indeed, SMD::hisCl animals showed increased reversal duration (Figures 5J and S5G) and lacked large-amplitude post-reversal turns (Figures S5H–S5J). SMDs therefore switch function depending on the long-timescale forward/reverse switch: during forward locomotion, they act as motor neurons, increasing head-bend amplitude and post-reversal turn amplitude, and driving head-casts; during reverse locomotion, in contrast, they do not affect head bending but rather promote state termination (Figure 5J). The latter suggests an interneuron-like role, consistent with SMDs synapsing onto interneurons in addition to their neuromuscular synapses (White et al., 1986).

We next asked whether these hierarchical relationships arise from intrinsic circuit properties or from proprioceptive feedback. To distinguish these possibilities, we examined SMD and DB activity during forward versus reverse command state in immobilized animals. As in behaving animals, mutually exclusive SMDD/V peaks were coincident with reversal command state termination (Figure S5K). Indeed, SMD::hisCl animals showed increased reverse command state durations (Figure 5K), confirming that this SMD function persists in the absence of behavior. Further, DB02 frequency and peak amplitude, SMDD peak amplitude (Figure S5L), and DB02 and SMD mean activity levels (Figure S2C) were reduced during reverse command states (Figure S5L), as in moving animals (Figure 5H). In conclusion, SMD and DB oscillations are hierarchically nested within the slower forward/reverse command cycle via circuit interactions.

**Figure 6:**
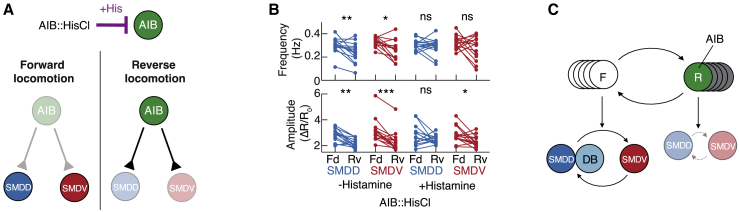
AIB Interneurons Orchestrate Upper-Level SMD Switch (A) Experiment design and hypothesis. (B) Frequency (upper) and average amplitude (lower; ΔR/R_0_ = normalized GCaMP/mCherry ratio) of SMD activity peaks in AIB::hisCl freely moving animals, ± histamine. ns, not significant; ^∗^p < 0.05, ^∗∗^p < 0.01, ^∗∗∗^p ≤ 0.001, Wilcoxon matched-pairs signed rank test. n = 14 (−histamine) and n = 15 (+histamine) animals. (C) Summary of investigated neuronal relationships. See also Figure S6 and Video S2.

We next sought to determine which neurons trigger the switch in SMD activity pattern. Among SMD’s pre-synaptic partners, AIB and RIM stood out as candidates, as both neurons are more active during reverse compared to forward locomotion (Gordus et al., 2015, Kato et al., 2015, Laurent et al., 2015, Luo et al., 2014, Piggott et al., 2011). We therefore hypothesized that AIB and/or RIM inhibit SMD activity during reversals (Figure 6A). We found that AIB inhibition abolished the forward/reverse locomotion modulation of SMDD frequency, SMDV frequency, and SMDD amplitude (Figure 6B). These results suggest that AIB is crucial for switching SMD neurons between high-activity and low-activity states (Figure 6C). Because RIM::hisCl animals lacked reversals, we took a genetic approach to RIM manipulation. Several studies have shown that RIM signals via tyramine to affect head oscillations and reversal behavior (Alkema et al., 2005, Donnelly et al., 2013, Pirri et al., 2009). We found a specific role for tyramine in SMDD frequency only (Figure S6). In summary, we found a major (AIB) and minor (tyramine, likely from RIM) role for two SMD inputs in switching SMD activity states at the uppermost hierarchy level. These findings are consistent with the hierarchy’s implementation via circuit interactions.

### Hierarchy Level II and III Interaction: Neuronal Phase Nesting

**Figure 7:**
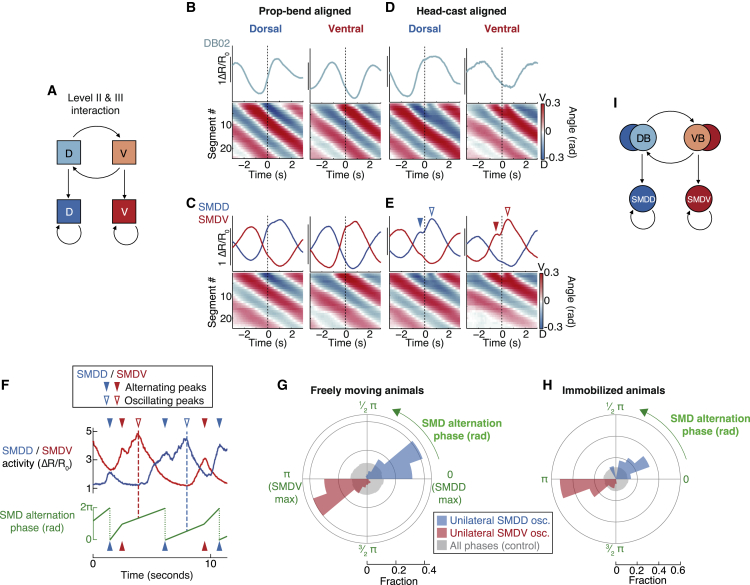
Hierarchy Level II and III Interaction: Phase Nesting of SMD Activity (A) Hierarchy levels investigated. (B–E) Trigger-averaged kymograms (lower) and neuronal activity traces (upper; ΔR/R_0_ = normalized GCaMP/mCherry ratio) from DB02 (B and D) and SMD (C and E) imaging, aligned to either propagated-bends without subsequent head-casts (B and C) or initial head-casts (D and E). Left to right, n = 669, 680, 303, and 101 (B and D) and n = 377, 360, 178, and 119 (C and E). In (E), activity peaks during propagated-bends (filled arrowheads) and head-casts (open arrowheads) denoted. (F) Example SMD activity time series illustrating SMD alternation phase measurement. Red and blue dashed lines indicate unilateral SMDD-only or SMDV-only oscillations, quantified in (G). (G and H) Fractional distributions of unilateral SMDD-only or SMDV-only oscillations binned according to SMD alternation cycle phase, in freely moving (G) or immobilized (H) animals. n = 196 (G, SMDD), 147 (G, SMDV), 57 (H, SMDD), and 23 (H, SMDV). p ≤ 10^−6^ for each SMD distribution in (G) and (H) indicates the probability that distributions are drawn randomly from the full data distribution shown in gray. Data in (B)–(E) and (G) pooled across 11 (SMD) and 10 (DB02) animals. Data in (H) pooled from 13 animals. (I) Summary of investigated neuronal relationships. See also Figure S7.

We next examined neuronal activity during the different head-bend types (Figure 7A). All three neurons showed oscillations synchronized with propagated-bends: DB02 and SMDD were active during dorsal propagated-bends, and SMDV was active during ventral propagated-bends (Figures 7B and 7C). SMDD and SMDV therefore showed alternating activity peaks with alternating dorsal/ventral propagated-bends (Figure 7C). During head-casts, DB02 did not show activity fluctuations beyond those during propagated-bends (Figures 7B and 7D; excepting a minority of especially posterior propagating head-casts, Figure S7A). In contrast, SMDs consistently showed oscillations during head-casts superimposed onto their propagated-bend activity (Figure 7E). In contrast to SMD's alternating activities during propagated-bends, SMD head-cast oscillations were strictly unilateral: SMDD oscillated during dorsal head-casts while SMDV remained inactive, and vice versa during ventral head-casts (Figures 7E and S7B–S7D; dotted lines in Figure 5C). SMDs therefore exhibited distinct activity signatures for different behaviors: SMDD/SMDV alternations during propagated-bends and unilateral SMDD-only or SMDV-only oscillations during head-casts.

**Figure 8:**
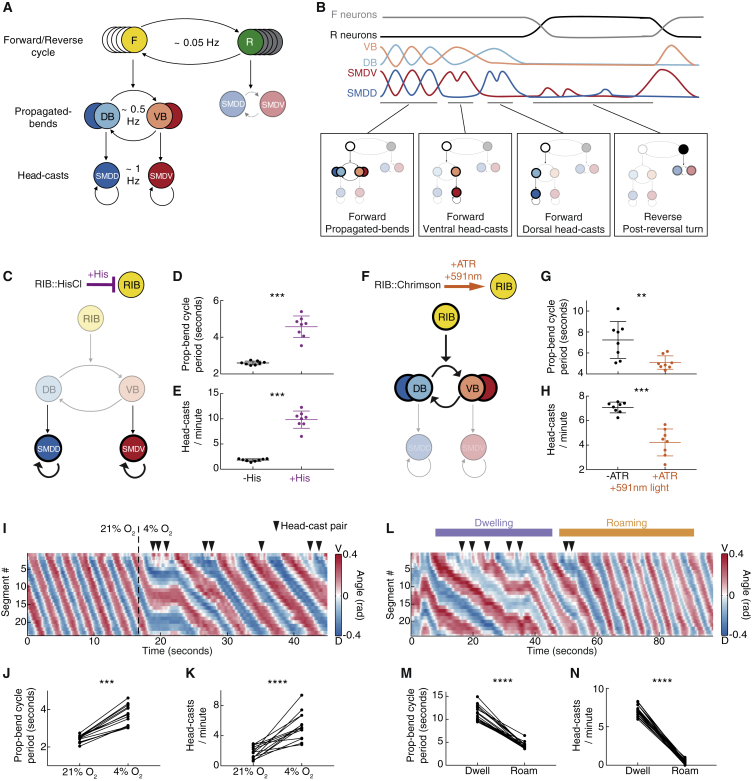
A Hierarchical Control Mechanism for Behavioral Flexibility (A) Neuron classes (F, forward-active; R, reverse-active) underlying each behavior. (B) Model traces (upper) and corresponding behavioral and neuronal hierarchical states (lower). (C) RIB inhibition model: longer propagated-bend cycle period results in increased head-cast frequency. (D and E) Propagated-bend cycle period (D) and head-cast frequency (E) in RIB::hisCl animals, mean ± SD n = 8 assays each. ^∗∗∗^p < 0.001, Mann-Whitney test. (F) RIB activation model: shorter propagated-bend cycle period results in decreased head-cast frequency. (G and H) Propagated-bend cycle period (G) and head-cast frequency (H) in RIB::Chrimson animals during yellow light exposure. n = 8 assays each. ^∗∗^p < 0.01, ^∗∗∗^p < 0.001, Mann-Whitney test. (I) Representative O_2_ downshift kymogram. (J and K) Propagated-bend cycle period (J) and head-cast frequency (K) 30 s pre- and post-O_2_ downshift. n = 14 assays. ^∗∗∗^p < 0.001, ^∗∗∗∗^p ≤ 0.0001, Wilcoxon matched-pairs signed rank test. (L) Example kymogram during dwelling to roaming switch. (M and N) Propagated-bend cycle period (M) and head-cast frequency (N) in dwelling or roaming animals. n = 19 assays. ^∗∗∗∗^p < 0.0001, Wilcoxon matched-pairs signed rank test. All data points show the mean of an experiment repeat with ∼20 animals each. See also Figure S8 and Videos S3 and S4.

We next asked whether these SMD activity signatures were phase-nested like their co-occurring behaviors. We quantified the phase of the SMDD/SMDV alternation cycle with SMDD peak – SMDV peak – SMDD peak corresponding to 0 – 1 – 2π rad (Figure 7F). This revealed that unilateral SMD oscillations were initiated at restricted phases (Figure 7G, compare with Figure 1F). This suggests that phase-nested SMD activity may be causal for phase-nested behavior; if so, phase-nested SMD activity should persist in the absence of behavior. Indeed, in immobilized animal recordings, SMD activity showed the same phase-nested relationship (Figure 7H). Further, these recordings revealed SMD activity relationships that likely underlie the proposed window of opportunity (Figures S7E and S7F, compare with S1H). The preservation of these dynamical relationships in immobilized animals is especially striking given that SMD activities fluctuate about one order of magnitude slower in immobilized versus moving animals (compare Figures S3E–S3H with S5A and S5B). Taken together, our data indicate that phase-nested neuronal dynamics are the cause, rather than consequence, of phase-nested behaviors (Figure 7I). Nested neuronal activity patterns are therefore a repeated dynamical motif of the *C. elegans* nervous system, which together constitute a hierarchical organization of neuronal activity and behavior across three timescales (Figures 8A and 8B).

### SMD-Head-Bend Synchrony Is Independent of RIA and GABA

SMD activity and head bending are strongly synchronized during propagated-bends in freely moving animals (Figures 5F and 7C), yet propagated-bends are largely unaffected by SMD inhibition (Figures 4B and S4A–S4D). The SMDs therefore likely act as followers during this behavior (Figure 7I), which our data suggest is driven by B-MNs (Figures 4B and S4A–S4D). In immobilized animals, however, DB01/02 showed no correlation with SMDD, indicating that other neuronal inputs, or proprioception, might entrain SMD oscillations with both B-MNs and propagated-bends.

We therefore examined two of SMD’s major inputs, RIA and RME. RIA is SMD’s most prominent presynaptic partner, and previous studies have shown a potent effect of RIA on SMD activity (Hendricks et al., 2012, Liu et al., 2018). We tested animals expressing tetanus toxin in RIA (Hendricks et al., 2012, Liu et al., 2018), thus blocking synaptic release, as well as RIA::hisCl animals (validated via imaging experiments, data not shown), and found no effect on the relationship between SMD activity and head bending (Figures S7G and S7H). RME activity showed strong, lateralized correlations with SMD activity in immobilized animals (Figure 3C) and has also been shown to functionally impact SMD activity via GABA signaling (Shen et al., 2016). *unc-25* mutants lacking GABA synthesis showed no defect in the relationship between SMD activity and head bending (Figure S7I). We therefore suggest that proprioception plays a prominent role in synchronizing SMD activity with propagated bends. Indeed, proprioception has been shown to modulate SMD activity (Yeon et al., 2018). Another possibility is that the B-MNs directly drive lateralized SMD activity in moving animals: B-MNs have also been shown to be proprioceptive (Wen et al., 2012) and are synaptically coupled to the SMDs via VB01.

### A Hierarchical Control Mechanism for Behavioral Flexibility

Phase nesting implies a mechanism for behavioral regulation: head-cast occurrence may be controlled by manipulating the propagated-bend oscillation speed, thereby widening or narrowing the head-cast window of opportunity. Propagated-bend oscillation speed correlated strongly with locomotion speed (Figure S8A); we therefore manipulated the RIB interneuron, a forward-active neuron previously implicated in locomotion speed control (Kato et al., 2015, Li et al., 2014). Indeed, RIB inhibition slowed the propagated-bend cycle and, consistent with our model, increased head casting (Figures 8C–8E). Conversely, optogenetic RIB activation accelerated the propagated-bend cycle and decreased head casting (Figures 8F–8H). To determine whether this antagonistic regulation occurs in more naturalistic conditions, we examined behaviors involving locomotion speed regulation. Off food, animals exposed to ambient O_2_ downshifts slow their locomotion (Zimmer et al., 2009), presumably to explore locally (Hums et al., 2016). Upon O_2_ downshift, slowed propagated-bend cycles were coupled with increased head casting (Figures 8I–8K and S8B; Video S3). On food, animals switch spontaneously between low-speed exploitative dwelling and high-speed explorative roaming states (Fujiwara et al., 2002). We found that roaming animals exhibited faster propagated-bend cycles and rarely head-casted compared to dwelling animals (Figures 8L–N, S8C, and S8D; Video S4). These results suggest context-dependent functions for head-casts, serving either exploration (off food) or exploitation (on food). They further implicate phase nesting as a mechanism for controlling behavioral output downstream of both sensory neurons and internal state regulators.

Video S3. O_2_-Induced Switch in Head-Bend Type, Related to Figure 8 Worm images and associated kymogram showing an example O_2_-downshift response, same as Figure 8I. Note the decrease in propagated-bend frequency and increase in head-cast frequency.

Video S4. Dwelling/Roaming Switch in Head-Bend Type, Related to Figure 8 Worm images and associated kymogram showing example dwelling and roaming periods, same as Figure 8L. Note the increase in propagated-bend frequency and decrease in head-cast frequency as the animal switches from dwelling to roaming.

## Discussion

Here, we showed that nested neuronal dynamics coordinate different timescale behaviors into a behavioral hierarchy in *C. elegans*. Our results conceptually reveal an organizational principle in which upper-level behavioral programs are represented by slow global dynamics spread across many neurons, while lower-level behaviors are represented by fast local dynamics in a few multi-functional neurons. Persistent phases of neuronal activity driving higher-level behaviors gate the occurrence of faster neuronal activity fluctuations driving lower-level behaviors. As a consequence, at lower hierarchy levels, neurons show dynamics that span multiple timescales incorporating those at the respective upper levels. Further, faster lower-level signals were exclusive to peripheral (i.e., motor) neurons, while slower upper-level signals permeated both central and peripheral neurons.

SMD activity changes span all three timescales. They show fast head-cast-driving oscillations (level III) superimposed onto a slower propagated-bend-correlated activity oscillation shared with the B-MN population (level II), which enables phase-nested behaviors (Figure 7). Upon switching to reverse locomotion at the interneuron level (level I), B-MNs become inactive, while SMDs change their activity patterns and execute completely different functions. SMDs are therefore highly multi-functional, executing both motor- and interneuron-like functions, in a manner strictly controlled by the hierarchical organization. Because neurons like the SMDs play roles at multiple hierarchical levels, our findings are best described as a hierarchy of neuronal dynamical states (Figure 8B), with each state consisting of particular activity patterns across many neurons, as opposed to a hierarchy in which individual neurons occupy distinct states and levels.

Some of the hierarchical relationships we describe can be straightforwardly linked to the worm’s known synaptic connectivity, such as descending interneuron control of motor neuron networks (Kawano et al., 2011, Xu et al., 2018). Other relationships, however, can be only partially explained by connectivity: while mutual inhibition between SMDD and SMDV could result from direct synapses between the two, phase nesting is a dynamical relationship that could be independent of connectivity. We nevertheless found that hierarchical nesting is a property of circuit interactions rather than feedback from proprioception or behavioral execution, as we could recapitulate nearly all of the nested relationships in paralyzed worms. Crucially, this indicates that the nervous system organizes its activities hierarchically, independent of behavior, and hierarchical behavior is inherited from this organization.

The hierarchy we describe (Figure 8A) is a rigid framework in which specific lower-level behaviors may only be accessed via switches at upper levels. For example, SMDV never exhibited head-cast activity during dorsal propagated-bends (when DB02 and SMDD were active); during this state, low-level ventral head-casts may only be executed by first switching the mid-level phase to ventral. This feature is specific to non-overlapping hierarchies, in which no lower-level state is connected to multiple upper-level states (Figure S8E) (Dawkins, 1976). This rigid framework is, however, accessible to additional neuronal control, via either sensory input or behavioral state (Figures 8I–8N); both of these can modulate the crawling-phase velocity of the animal and therefore the window of opportunity for head casting. Given the ubiquity of neuronal activity oscillations as well as hierarchically organized behavior, the circuit interactions and control principles we describe may be relevant for other species.

We also examined an even longer timescale of behavior above the forward-reverse switch: roaming and dwelling behavioral states. In contrast to the strict, all-or-none relationships of the motor hierarchy, actions within roaming and dwelling are not exclusive to those states but rather show differences in frequency: dwelling exhibits high frequencies of forward-reverse transitions and head-casts, while roaming consists of long stretches of propagated-bend forward movement with low head-cast and reversal frequencies (Figures 8I–8N; Gallagher et al., 2013). We therefore suggest that such longer-timescale behavioral states, which rely on neuromodulation (Ben Arous et al., 2009, Flavell et al., 2013), are best described by a less restrictive overlapping hierarchical model, in which lower-level states may be shared by different upper-level states (Figure S8E; Dawkins, 1976). Tinbergen’s non-overlapping hierarchical model for stickleback behavior included longer-lasting states typically controlled by neuromodulators, such as a reproductive instinct (Tinbergen, 1951). The non-overlapping hierarchy we describe (Figure 8A) is more in line with recent studies describing hierarchies at the level of motor actions (Berman et al., 2016, Duistermars et al., 2018, Glaze and Troyer, 2006, Gomez-Marin et al., 2016, Marques et al., 2018, Wiltschko et al., 2015). A primary benefit of hierarchical behavior may be to coordinate the activities of motor neurons that target overlapping body parts, in order to prevent interfering actions. In contrast to the rigid framework studied here, longer-lasting behavioral states could gain flexibility by re-utilizing a combinatorial set of actions.

The strict, nested relationships we uncovered bear striking resemblance to neuronal dynamics underlying multi-timescale rodent behaviors. For example, CPGs driving whisking and breathing appear to interact in a hierarchical manner: breathing resets the whisking phase, but not vice versa, and a unidirectional anatomical pathway from the breathing CPG to the whisking CPG has been described (Moore et al., 2013). Such a phase-resetting mechanism may also explain the impact of B-MNs on SMD activity, whereby B-MN oscillations may drive the SMDs into particular phases that restrict their unilateral oscillation and therefore head-casts. Further, basal ganglia circuits in mice show independent coding of different timescales during learned action sequences (Jin et al., 2014), suggesting a hierarchical “chunking” with theoretical advantages compared to serial sequencing (Jin and Costa, 2015). Our study demonstrates how circuits acting on different timescales can interact to implement hierarchical behavior and could therefore provide a starting point to search for similar circuit interactions in other organisms.

The motor neuron dynamics showed a strong dependence on oscillation phase (Figures 7G and 7H). Oscillation phase is crucially important for the effect of motor neurons during insect locomotion (Fayyazuddin and Dickinson, 1999, Sponberg and Daniel, 2012) and has been proposed to act as a window for integrating inputs in flies (Gupta et al., 2016) and mammals (Buzsáki and Draguhn, 2004, Yuste et al., 2005). While forward- and reverse-state switches in *C. elegans* appear to be stochastic rather than oscillatory (Roberts et al., 2016), non-oscillatory phase coding has been demonstrated in the bat (Eliav et al., 2018). It has also been suggested that CPG circuits may foretell principles of cortical circuits (Yuste et al., 2005). Throughout the mammalian brain, longer-timescale oscillations spread across larger neuronal populations influence more local, shorter-timescale oscillations in a hierarchical manner (Buzsáki et al., 2013). Coupling between these nested oscillations could serve myriad functions (Hyafil et al., 2015); for example, the widening and narrowing of an alpha-oscillation window of opportunity has been proposed to enable gamma-oscillatory communication between neuronal populations (Hahn et al., 2019). Further, comprehension of speech, a naturally hierarchical stimulus, involves the emergence of neuronal dynamics across timescales, with spatially dissociable representations for distinct frequencies (Ding et al., 2016). Our work demonstrates that such hierarchically nested dynamics can indeed organize neuronal activity patterns, and, consequently, animal behavior, across timescales.

In conclusion, nested neuronal activity patterns implement a three-level hierarchy of behavior across timescales in *C. elegans*. Neuronal activity dynamics on different timescales are ubiquitous in neuroscience; we therefore speculate that such hierarchically nested activity patterns may be a common mechanism for organizing and regulating neuronal dynamics across timescales.

## STAR★Methods

### Key Resources Table

**Table:** 

REAGENT or RESOURCE	SOURCE	IDENTIFIER
**Experimental Models: Organisms/Strains**		

*C. elegans*: Strain ZIM958: *lite-1 (ce314)*	This study	N/A
*C. elegans*: Strain ZIM1466: *lite-1 (ce314); mzmEx877[Pnlr-1(−150;-1)::HisCl::SL2::mCherry* ]*; mzmIs52[Punc-31::NLSGCaMP6f]*	This study	N/A
*C. elegans*: Strain ZIM1564: *lite-1 (ce314); mzmEx929[Punc-7 s::CreVDH;Pmyo-3::mCherry]*	This study	N/A
*C. elegans*: Strain ZIM1725: *lite-1 (ce314); mzmEx1018[Punc-7 s::CreVDH;Pmyo-3::mCherry]*	This study	N/A
*C. elegans*: Strain ZIM1418: *lite-1 (ce314); mzmEx858[Pflp-22::DIO-HisCl::SL2::mCherry;Pelt-2::NLSdsRedNLS]*	This study	N/A
*C. elegans*: Strain ZIM1473: *lite-1 (ce314); mzmIs28[Punc-17beta::HisCl::SL2::mCherry]*	This study	N/A
*C. elegans*: Strain ZIM1628: *lite-1 (ce314); mzmEx877[Pnlr-1(−150;-1)::HisCl::SL2::mCherry*]*; mzmEx929 [Punc-7 s::CreVDH;Pmyo-3::mCherry*]*;mzmIs52 [Punc-31::NLSGCaMP6f]*	This study	N/A
*C. elegans*: Strain ZIM1748: *lite-1 (ce314); mzmEx877[Pnlr-1(−150;-1)::HisCl::SL2::mCherry*]*; mzmEx1018 [Punc-7 s::CreVDH;Pmyo-3::mCherry*]*;mzmIs52 [Punc-31::NLSGCaMP6f]*	This study	N/A
*C. elegans*: Strain ZIM1562: *lite-1 (ce314); mzmEx877[Pnlr-1(−150;-1)::HisCl::SL2::mCherry*]*; mzmEx858 [Pflp-22::DIO-HisCl::SL2::mCherry;Pelt-2::NLSdsRedNLS* ]*;mzmIs52 [Punc-31::NLSGCaMP6f]*	This study	N/A
*C. elegans*: Strain ZIM1574: *lite-1 (ce314); mzmEx877[Pnlr-1(−150;-1)::HisCl::SL2::mCherry*]*; mzmIs28 [Punc-17beta::HisCl::SL2::mCherry];mzmIs52 [Punc-31::NLSGCaMP6f]*	This study	N/A
*C. elegans*: Strain ZIM1658: *lite-1 (ce314); mzmEx981[Punc-17beta::NLSGCaMP6f]*	This study	N/A
*C. elegans*: Strain ZIM1467: *lite-1 (ce314); mzmEx882[Punc-7S::CreVDH;Pflp-22::DIO-mCherry;Pflp-22::DIO-GCaMP6Fopt]*	This study	N/A
*C. elegans*: Strain ZIM2122: *lite-1 (ce314); mzmEx1268[Pinx-1::hisCl; Pflp-17::mCherry]*	This study	N/A
*C. elegans*: Strain ZIM2105: *lite-1 (ce314); tdc-1 (n3419); mzmEx882[Punc-7S::CreVDH; Pflp-22::DIO-mCherry;* *Pflp-22::DIO-GCaMP6Fopt]*	This study	N/A
*C. elegans*: Strain ZIM2106: *lite-1 (ce314); lgc-55(n4331); mzmEx882[Punc-7S::CreVDH; Pflp-22::DIO-mCherry; Pflp-22::DIO-GCaMP6Fopt];*	This study	N/A
*C. elegans*: Strain ZIM2124: *lite-1 (ce314); yxEx696 [Pglr-3::TeTx::mCherry; Punc-122::dsRed]; mzmEx882[Punc-7S::CreVDH; Pflp-22::DIO-mCherry; Pflp-22::DIO-GCaMP6Fopt]*	This study	N/A
*C. elegans*: Strain ZIM2120: *lite-1 (ce314); mzmEx1262 [Pglr-3::hisCl::SL2::mCherry; Pflp-17::mCherry]; mzmEx882[Punc-7S::CreVDH; Pflp-22::DIO-mCherry; Pflp-22::DIO-GCaMP6Fopt]*	This study	N/A
*C. elegans*: Strain ZIM2128: *lite-1 (ce314); unc-25 (e156); mzmEx882[Punc-7S::CreVDH; Pflp-22::DIO-mCherry; Pflp-22::DIO-GCaMP6Fopt];*	This study	N/A
*C. elegans*: Strain ZIM1749: *lite-1 (ce314); mzmEx1041[Psto-3::HisCl::mCherry;Punc-122::GFP]*	This study	N/A
*C. elegans*: Strain ZIM1563: *lite-1 (ce314); mzmEx928[Psto-3::Chrimson::mCherry;Punc-122::GFP]*	This study	N/A

### Lead Contact and Materials Availability

All unique/stable reagents generated in this study are available from the Lead Contact (M.Z., manuel.zimmer@univie.ac.at) with a completed Materials Transfer Agreement.

### Experimental Model and Subject Details

All experiments were performed in *lite-1 (ce314)* animals to reduce light responses (Liu et al., 2010). Young adult hermaphrodites were used for all experiments. Worms were maintained using standard methods (Brenner, 1974) and grown at 20°C on nematode growth media (NGM) plates, which were seeded with *Escherichia coli* OP50 as a food source. A detailed list of all transgenic strains used is provided in the Key Resources Table and Table S1.

### Method Details

#### Population behavior assays

For on-food assays (Figures 8G, 8H, 8L–8N, S8C, and S8D), ∼20 animals (young adults, 0 eggs to 1 row of eggs) were transferred to a 15cm NGM assay plate seeded with OP50 (previously grown for 16 h at room temperature). For off-food assays (all other behavior experiments) animals were picked onto a food-free NGM plate, immersed in 1mL S-basal to remove transferred food, and picked again onto a food-free 15cm NGM assay plate. Data in Figure 1C are combined from an equal number of on- and off-food experiments, because head-casts make up the majority of bends on-food and propagated-bends make up the majority off-food. Data in Figure 1D and Figures S1B-S1D, S1G, and S1H are taken only from animals off-food to enable comparison within a single condition. Data in Figures S1E–S1G are taken from Ca^2+^ imaging experiments, which showed a higher number of head-cast episodes with more 4-6 head-casts and which had dorsal/ventral sides annotated, unlike animals recorded in behavior assays. In both on- and off-food cases, a 36mm x 36mm assay arena was delineated by Whatman paper soaked with 20mM CuCl_2_, a repellent used to prevent worms from leaving the arena. A custom transparent plexiglass gas flow device (Hums et al., 2016) of 39mm x 39mm x 0.7mm was placed on top of this arena, through which 21% O_2_ was delivered at 25mL/min via a static gas mixer connected to mass flow controllers (Vögtlin Instruments), operated by custom written LabVIEW scripts (National Instruments). For off-food assays, 21% O_2_ was switched to 4% O_2_ to examine sensory responses; for on-food assays, 21% O_2_ was required to observe significant roaming periods. Arenas were illuminated with red LEDs and movies were recorded at 10fps on 5 megapixel CMOS cameras (Teledyne DALSA) with a pixel resolution of 0.0129 mm/pixel. Animals were allowed to acclimate for either 5min (off-food) or 1 h (on-food) prior to recordings.

For histamine assays, prior to picking onto the assay plates, animals were incubated for 30-45min on plates with NGM agar including either 20mM histamine (+His; histamine dihydrochloride, Sigma-Aldrich) or an equal volume of water (-His) and seeded with OP50 across the entire agar surface to enhance histamine uptake. Recordings were performed on +His and -His assay plates. For optogenetics assays, animals expressing the optogenetic activator Chrimson (Klapoetke et al., 2014) were grown on NGM plates seeded with OP50 plus either 100 μM all-trans retinal (ATR) or an equal volume of ethanol lacking ATR (Control). One day prior to assays, L3 and L4 animals were picked onto fresh ATR or Control plates. After 30min without light, constant 591nm light was delivered, via a custom-made ring of LEDs above the assay plate, at ∼45 μW/mm^2^ for 30min.

Movies were analyzed using custom image processing and tracking code in MATLAB (Mathworks), built upon previously published scripts (Ramot et al., 2008). Briefly, worms were detected by gray level thresholding, and trajectories were found by adjoining nearby centroid coordinates in adjacent frames. These centroid data were used for speed, angular speed, reversal, and heading change quantifications. To measure bend angles, worm images were analyzed as described in (Hums et al., 2016). Briefly, binarized worm images were skeletonized to produce splines tracing the midline of each worm. Splines were smoothed and divided into 25 equally spaced body segments. Head versus tail positions were determined by direction of centroid movement. Angles were measured between adjacent segments as in Figure 1A to produce 24 angle measurements for each frame in each worm’s trajectory. Power spectra in Figures S3E–S3H, S4C, and S5A–S5C were calculated using the MATLAB (Mathworks) function fft on the timeseries concatenated across experiments. For experiments in which manipulations caused a loss of animal movement, it was important to exclude time-averaged background subtraction prior to gray level thresholding, and to assign head position manually. Ventral and dorsal were indistinguishable in these lower-resolution recordings, so angle sign was assigned randomly; all panels distinguishing ventral and dorsal directions made use of higher-resolution movies recorded along with Ca^2+^ imaging (see below).

#### Ca^2+^ imaging in immobilized animals

For immobilized Ca^2+^ imaging whole-brain and whole-nervous system experiments, transgenic adult *C. elegans* (1 day after larval L4 stage, 0-10 eggs) expressing genetically-encoded calcium indicator NLS-GCaMP6f (Chen et al., 2013) panneuronally (using the *Punc-31* promoter) were recorded as described previously (Kato et al., 2015) with following modifications. Previously, we found that forward command states occasionally terminate in quiescence periods (Kato et al., 2015, Nichols et al., 2017). Thus, to increase forward command state occurrence, we expressed the *Drosophila* hisCl channel (Pokala et al., 2014) in the quiescence-promoting neuron RIS (Nichols et al., 2017, Turek et al., 2013) with the *Pnlr-1* (Gendrel et al., 2016, Haklai-Topper et al., 2011) promoter. To silence the SMDs, hisCl was expressed specifically in these neurons using a Cre-lox strategy. To this end, we expressed Cre with the P*unc-7S* promoter (Starich et al., 2009) and the *HisCl::SL2::mCherry* construct within a double inverted open reading frame (DIO) with the *Pflp-22* promoter (Kim and Li, 2004). SMD inhibition was confirmed using the mCherry marker co-expressed with HisCl while co-recording GCaMP from the SMD neurons; animals showing residual SMD activity (∼50% of animals) were excluded from further analysis. Residual SMD activity often observed in mCherry-positive neurons (data not shown) may explain residual head-casting in SMD::hisCl animals (Figure 4B). Body MNs were silenced by expressing hisCl and mCherry in VNC cholinergic motor neuron classes AS, DA, DB, VA, VB with promoter *Punc-17β* (Charlie et al., 2006); this promoter is referred to as VNC_ACh_ throughout. While we could not use the mCherry marker to unambiguously identify DB01 and DB02 in these animals, we confirmed that all mCherry-positive neurons in the retrovesicular ganglion (where DB01 and DB02 nuclei reside) showed little to no activity.

Immobilized Ca^2+^ imaging experiments were performed with custom-made microfluidic two-layer PDMS devices as previously described (Kato et al., 2015, Schrödel et al., 2013), with modifications. In addition to the curve in the worm channel used to align worms laterally (Cáceres et al., 2012), a straightening of the channel was followed by a second curve designed to fit a young adult worm. This curve was designed to align the animal’s head and tail, thus reducing the necessary imaging area to record the activity of all neurons in the *C. elegans* body; it also contains a narrowing in order to keep the animal’s head in place. A technical drawing of the design is provided in Figure S2A.

As previously described (Hums et al., 2016, Zimmer et al., 2009), the worm channel of the microfluidic device was connected to a syringe containing nematode growth medium (NGM) buffer with 1 mM tetramisole and 20 mM histamine (his-tet-NGM). All components were connected using Tygon tubing (0.02 in ID, 0.06 in OD; Norton) or polyethylene tubing (0.066 in ID, 0.095 in OD; Intramedic) using 23G Luer-stub adapters (Intramedic). Constant gas delivery at 21% O_2_ at a flow rate of 50ml/min was regulated with a gas mixer attached to mass flow controllers (Vögtlin Instruments) that mixed oxygen and nitrogen from pressurized gas tanks, using LabView software.

Well-fed worms were transferred onto NGM agar plates seeded with OP50 *E. coli* mixed with 20 mM histamine to feed on for 30-45 min. To rid them of bacteria covering the body, the animals were transferred into a drop of his-tet-NGM on a food-free NGM agar plate and then onto a second NGM plate with his-tet-NGM buffer. A vacuum was manually applied with the syringe to suck up individual animals into Tygon tubing; this tubing was subsequently reconnected to the worm inlet to arrange the worm in the curved channel. The fluorescence values were recorded 5 min after loading; the illumination and piezo stage were switched on 2-3 min before acquisition start. Animals were imaged at 21% O_2_ for 30 min.

High-resolution data of neuronal activity in the head ganglia (whole-brain recordings) was acquired with an inverted UltraViewVoX spinning disk confocal microscope (PerkinElmer) using an EMCCD camera (C9100-13, Hamamatsu) and a 40x 1.3 NA EC Plan-Neofluar oil-immersion objective (Zeiss). The volume spanning the animals’ head ganglia was recorded in 13-15 2 μm z-planes, each illuminated for 10ms to record GCaMP6f fluorescence, resulting in acquisition rates of 1.55 - 2.23 volumes/sec. High-speed, high-resolution data of SMD neuronal activity in immobilized animals was acquired to confirm that the acquisition rate of whole-brain recordings was sufficient to capture SMD dynamics. This was achieved using with an inverted spinning disk confocal microscope (Observer Z1, Zeiss with Yokogawa CSU-X1 spinning disk) with a 40x 1.2 NA LCI Plan-Apochromat multi-immersion objective (Zeiss) with an EMCCD camera (Evolve 512, Photometrics). To obtain data with high temporal resolution, we recorded single planes illuminated for 20ms, resulting in an acquisition speed of 50 Hz. Whole-nervous system neuronal activity was recorded using an inverted fluorescence microscope (Observer Z1, Zeiss) with a 25x 0.8 NA LCI Plan-Neofluar multi-immersion objective (Zeiss) and recorded with a scientific complementary metal-oxide-semiconductor (sCMOS) camera (pco.edge 4.2, PCO) using Visiview software (Visitron Systems). The nervous system was recorded in 30 1 μm z-planes illuminated each for 10ms, resulting in an acquisition rate of 3.0303 volumes/sec. To increase the contrast and resolution of the image data in the immobilized whole-nervous-system recordings exclusively, we used a deconvolution algorithm (classic maximum likelihood estimation) using Huygens software (signal/noise ratio, 8; automatic background estimation; iterations, 40; quality change stopping criterion, 0.1). After deconvolution, the neuronal activity traces extracted from immobilized whole-nervous-system recordings were compared to “ground truth” high-resolution whole-brain recordings acquired with spinning disk confocal microscopy in order to assess the quality of the whole-nervous-system data and to ensure that the deconvolution procedure did not introduce artifacts. Importantly, deconvolution was performed only for whole nervous system recordings in immobilized worms because a standard spinning disk confocal microscope (Yokogawa CSU-X1 scanning head) does not provide the needed field of view. Head-tail multi neuron imaging and all imaging on freely moving animals did not include image deconvolution post processing steps.

#### Simultaneous imaging of neuronal activity and behavior

Transgenic young adult worms (0-6 eggs), expressing GCaMP6f and mCherry in specific neurons of interest, were placed onto a foodless NGM agar plate, allowed to crawl away from food, then picked onto another foodless NGM agar plate. Then, as in (Collins and Koelle, 2013), a ∼40mm x 40mm chunk of agar surrounding the worm was removed and covered with a 45mm x 50mm #1.5 coverglass, so that the worm was between coverglass and agar. Under these conditions worms move slower but with qualitatively normal body shapes and behavior. The coverglass setup was placed into a motorized stage with associated controller (MS-2000-PhotoTrack, Applied Scientific Instrumentation). After a 5min acclimation period including at least 2min excitation light exposure, each worm was recorded for 8min. Images were acquired using an inverted compound microscope (Ziess Axio Observer.Z1) with two Charge-Coupled Device cameras (Evolve 512, Photometrics). A CoolLED pE-2 excitation system provided dual wavelength excitation light (470 and 585 nm) with an ET-EGFP/mCherry filter set (59022x, Chroma) and dichroic (59022bs, Chroma). A 63x oil objective (Zeiss Plan-Apochromat, 1.4 NA) was used to stream unbinned single-plane fluorescence images at 33ms exposure time, resulting in ∼30 Hz imaging frame rate, with Visiview software (Visitron Systems GmbH). Sparse drivers were used to enable stable GCaMP expression specifically to neurons of interest (and few others), a precaution necessary to ensure the unambiguous identification of specific neurons of interest and the subsequent extraction of their neuronal signals. The neuron(s) of interest were re-centered onto the objective using the system described in (Faumont et al., 2011): a dichroic mirror (620 spxr, Chroma) directed the high-wavelength portion of the mCherry emission to a four-quadrant photomultiplier tube (Hamamatsu). Remaining emission light was split by a DualCam DC2 cube (565 lpxr, Photometrics) to each CCD camera, one for mCherry (641/75nm, Brightline) and one for GCaMP (520/35nm, Brightline). Simultaneous behavior recordings were made under infrared illumination (780nm) using a CCD camera (Manta Prosilica GigE, Applied Vision Technologies) at 4x magnification and 100ms exposure time. Approximately 8 min of data were acquired for each animal.

#### Data Analysis

##### Neuronal time series extraction from immobilized pan-neuronal imaging experiments

As described before (Kato et al., 2015), neuronal activity traces were obtained by tracking the intensity maxima in each volume over time and calculating the single-cell fluorescence intensities (F). F_0_ was calculated as the mean fluorescence intensity across the trial. After background subtraction, ΔF/ F_0_ was calculated for each neuron. ΔF/F_0_ neural traces were then detrended to correct for bleaching in a two-step procedure which gave qualitatively better results compared to the previously described method (Kato et al., 2015). First, we performed and exponential fit to each trace, followed by fitting a single exponential function to peaks of the neural traces. This second bleach-correction step (referred to as detrending), occasionally drastically over-corrected and resulted in distorted neuronal traces in a small number of instances. Thus, we calculated the relative change in variance of each trace after the second detrending step (var(post−detrending)−var(pre−detrending)/var(pre−detrending)) as a measure of distortion. The distribution of these measures across all neurons exhibited a long tail and instructed us to determine a relative change of 8 as a cutoff for distorted traces. For neural traces above the variance change cutoff (3.31% of the neuronal traces analyzed), we resorted to the single bleach-corrected version of the trace.

##### Neuronal identification (pan-neuronal imaging)

In whole-brain Ca^2+^ imaging data, the activity of 113-129 neurons was detected in the head ganglia, which corresponds to 49.7 - 66.15% of expected neurons. The rest likely were constitutively inactive and thus showed very low fluorescence levels; we further cannot exclude that the label is not expressed in a small fraction of neurons. In whole-nervous system Ca^2+^ imaging data, 103-129 neurons were detected across the whole body (39.4 - 42.7%).

The identification of the neurons of interest was done according to their activity patterns, anatomical location (https://www.wormatlas.org/) and our experience with red fluorophore expression in specific marker lines reported in previous recordings (Kato et al., 2015, Nichols et al., 2017, Skora et al., 2018). In addition, the SMD neurons were identified by driving worm codon-optimized mCherry (wCherry) (Kawano et al., 2011) expression with the *Pflp-22* and *Punc-7 s* promoters; henceforth, we disambiguated the uncertain cell class identity of these neurons reported in previous studies (SMDD versus SMB or RMF)(Kato et al., 2015, Schrödel et al., 2013). B-MNs along the VNC were identified with the *Pacr-5* promoter (Winnier et al., 1999); PDA was identified with a Cre-lox strategy using the P*flp-7*::*Cre* and P*nmur-1*::*DIO-mCherry* constructs (Kim and Li, 2004, Maier et al., 2010). We denoted # cases in which reliably observed activity patterns in distinct locations could not be unambiguously assigned to neuronal identities; their neuronal identities and possible alternatives are shown in brackets: URY (URA, IL1), RMD (SAA, RIA, SIB, RIH), SIB (SIA, RMD, RMH).

B-MN IDs were determined by a combination of position, activity, and specific marker line information. We consistently observed four neurons in the retrovesicular ganglion (RVG) with heightened activity during forward command states; we labeled the anterior-most neuron VB02 and the posterior-most neuron DB01, in agreement with previous IDs (https://www.wormatlas.org/). Of the remaining two neurons, we consistently found one with activity patterns like DB01 and VB02 (i.e., slow rises following AVA fall and activity plateaus throughout forward command states) and one with more spike-like, unpredictable activity transients. We used P*ceh-12::mCherry* to label VB neurons (Von Stetina et al., 2007) while recording and found that the latter neuron is VB01; the former is therefore DB02. Other B-MNs are reported to occur in a specific pattern along the VNC: DB03 and VB04 are posterior to the RVG and separated by cell bodies of other neuron classes, and posterior to VB04 is a repeating pattern of consecutive (i.e., uninterrupted by other cell bodies) VB/DB pairs followed by single VB neurons. We used P*acr-5::mCherry* to label all B-MNs and their processes and confirmed this 2-1-2-1 pattern. This marker also showed that in VB/DB pairs, the VB neuron is always anterior (DB but not VB neurons have commissures that cross to the dorsal side). One exception to this rule was the posterior-most pair: 11/20 animals showed DB07 anterior to VB11. In whole-nervous-system recordings, we found the expected number of neurons in the expected anatomical pattern showing heightened activity during forward command states in all recordings. This allowed us to assign IDs to all such neurons except DB07 and VB11; we labeled the anterior of this pair DB07a and the posterior VB11p.

##### Principal Component Analysis (PCA; immobilized pan-neuronal imaging)

Our previously reported neuronal activity studies in freely moving animals showed that behavioral parameters like motor state, crawling speed and turning strength can sometimes better be decoded either from the time derivatives or original Ca^2+^-imaging traces, depending on neuronal class (Kato et al., 2015). Unlike in our previous work, where we performed PCA only on the time derivatives (Kato et al., 2015, Nichols et al., 2017, Skora et al., 2018), we here performed PCA on both original Ca^2+^-imaging traces plus their derivatives, thus each neuron is represented by two variables (rather than one) where each recording frame is one observation. This procedure is less biased as it captures both instantaneous Ca^2+^-levels as well as the dynamics of each neuron. First, the time derivatives of ΔF/F_0_ of the detrended neuronal activity traces were calculated using the total variation regularized differentiation method (Chartrand, 2011). The neural traces were normalized so that each trace and its time derivative had equal variance. PCA was performed on the detrended ΔF/F_0_ neural traces and their time derivatives simultaneously using the MATLAB (Mathworks) pca function, which also calculated variance explained for each PC. For visualization purposes, a 10-sample sliding average filter was applied to the PCA-phase plot trajectories. Command state coloring of the phase plot trajectories was performed similarly as described previously (Kato et al., 2015). Shortly, AVA, SMDD and SMDV activities were used to assign behavior state commands to population neuronal activity. For this, RISE, HIGH, FALL and LOW phases were identified for these neurons as follows: RISE and FALL phases were defined as time points when their time derivative was greater than a small positive threshold or smaller than a negative threshold, respectively. The remaining time points were assigned to HIGH and LOW phases based on behavioral state order and a threshold. The time points in the trajectory of neuronal activity were then colored by AVA phase (AVA rise and high as reversal, fall as a post-reversal turn, and low as forward command)(Figures 2C and S2B). Note that AVA falls were coupled to either SMDV or SMDD rises and these were mutually exclusive in 100% of all detected AVA falls (Figure S5K).

##### Command state identification, duration quantification (immobilized pan-neuronal imaging)

Forward command states were inferred from the activity of interneuron AVA, as previously described (Kato et al., 2015). Shortly, time points of falling Ca^2+^ transients and low intensity AVA Ca^2+^ signals were defined as forward command states. Time points of rising Ca^2+^ transients and high intensity Ca^2+^ signals were defined as reversal command states.

##### Mean activity difference quantification (pan-neuronal imaging)

In order to assess whether neuronal activity levels are modulated by the forward/reverse command state, we calculated for each neuron the mean ΔF/F_0_ of the Ca^2+^ imaging neural activity traces in all forward and reverse command states, separately, and calculated the difference. As for the peak frequency quantification (see below), all neurons identified in at least three recordings were taken into account. We determined which neurons showed a significant difference between their mean activity levels in the forward versus reverse command states with a paired t test (p values reported in Table S2).

The distribution of forward command state duration was bimodal (data not shown) and states shorter than 50 s rarely allowed for more than one fluctuation per state. In order to avoid a bias in our analysis because of lack of data, forward command states shorter than 50 s were excluded from the analysis. Frequencies were calculated as the number of maximum peaks within each forward command state divided by the duration of each forward command state for each neuron. Instances where a neuron was identified but no peaks were detected were counted as zero.

##### Peak frequency quantification (immobilized pan-neuronal imaging and freely moving imaging)

Peak detection was used to quantify neuronal activity data shown in Figures 3, 4, 5, 6, 7G, 7H, S2D, S2E, S3C, S3D, S5D, S5L, S6, and S7E–S7I. For Figures 3, 4, S2D, and S2E only, peaks were detected on derivative timeseries: for all neurons identified in at least three recordings, we obtained their smoothed time derivatives with the total variation regularized differentiation method (Chartrand, 2011). For all peak detections, derivative and non-derivative, we took the following approach. We detected local maxima and minima in each trace: maximum peaks were found as maximal values that were preceded and followed by minima values separated from the maximum by an amplitude difference of at least delta, a key parameter. Peak detection involves a tradeoff between false positives and false negatives; the parameter delta determines how liberal (more false positives) or conservative (more false negatives) the peak detection is. We developed a simple method to automatically determine a near-optimal delta value (i.e., minimizing both false positives and false negatives) for each neuronal activity trace. We first perform peak detection with a wide range of deltas that spans from too liberal (many false positives, due to detection of noise) to too conservative (many false negatives, large amplitude peaks not detected). Then, we plot the number of peaks detected as a function of delta. The shape of this curve varies depending on the activity trace, but often resembles a piecewise linear function of two parts. At low delta values, the slope is high: the number of peaks detected by low delta values is dominated by false positives due to noise, and in this case, small increases in delta cause large decreases in the number of peaks detected. At high delta values, the slope is low: the peaks detected at high delta values are only real (i.e., large amplitude) ones, and in this case, small increases in delta typically result in little to no change in the number of detected peaks. Therefore, the slope of the curve at high delta values is much closer to 0 than at low delta values. In the ideal case, the change point between these two lines is clear, giving an optimal delta value at which the numbers of both false positives and false negatives are minimized. We determined this point using the MATLAB (Mathworks) function *findchangepts*, with the ‘linear’ option; this outputs the index of the curve at which both the mean and slope change most abruptly.

This method matched our hand-chosen “ground truth” detected peaks remarkably well for several traces, and performed much better than choosing a single delta value for all traces in a dataset. However, this method remains dependent on two parameters. First, all peak detection methods depend strongly on the degree to which the data are smoothed. Second, the range of deltas tested determines whether or not this method will succeed. If the delta range is dominated by deltas that are too low or too high, it will not find an adequate changepoint, or the changepoint will be too liberal or too conservative for all traces. We found that a single range of deltas works well for all data in a particular noise (e.g., sampling rate, degree of smoothing) and amplitude (e.g., derivative or non-derivative) regime. We also found that a good starting point to determine the delta range for a particular trace was to use the standard deviation, setting this value as the maximum of the delta range, while setting the minimum and interval between deltas as the standard deviation divided by 100. In this work, for each of four different types of datasets, we used a different delta range, as follows. For peak detection on derivatives from immobilized worm recordings (Figures 3, 4, and S2), the delta range went from 10^−4^ to 0.03 in steps of 5x10^−5^. For peak detection on raw traces of immobilized worm recordings at 1.5-3 Hz (Figures 7H, S3C, S3D, S5L, and S7F), the delta range went from 0.001 to 0.5 in steps of 0.001. For peak detection on raw traces of immobilized worm recordings at 50 Hz (Figures S3C and S3D), the delta range went from 0.08 to 0.3 in steps of 0.0005. For freely moving imaging recordings (Figures 5, 6, 7, and S5–S7), the delta range went from 0.001 to 1 in steps of 0.001. For peak detection on derivatives, which gave better results across all neurons in brain-wide recordings than peak detection on raw traces, two additional steps were made: (1) peaks with amplitudes less than 0 were excluded, as these are changes in the slope of a fall rather than rises; (2) the second of two subsequent maxima was only included if the intervening minimum fell below 0. If the intervening minimum did not fall below 0, the two peaks correspond to changes in slope rather than individual calcium peaks, and the second peak was therefore excluded.

##### Cross-correlation analysis (immobilized pan-neuronal imaging)

Covariograms were calculated for all neurons that could be reliably identified across at least three immobilized worm recordings. Covariograms report the frequency, relative to chance levels, of a “target” neuron’s peak at different time delays relative to a “reference” neuron’s peak. We found covariogram analysis superior to standard cross-correlation analysis for our sparse data. For example, neurons often showed only one or two activity transients per forward command state. Standard cross-correlation on sparse data would report a high value at a given lag for two neurons showing few activity transients each, despite having independent signals. For example, a toy dataset of two signals, one oscillatory and one with a single step or few step signals would result into a strong cross correlation signal, which would otherwise vanish, if the time-series could be expanded to capture many sparse signals, which often is experimentally unfeasible. Averaging max cross-correlation values across forward states would therefore result in high correlations for neurons showing sparse activity. Covariograms, by contrast, take into account the number of peaks detected, thus enabling statistical testing to exclude cases with few peaks from significance.

Covariograms were computed as described in ref. (Brody, 1999) with some minor adjustments to account for variations in forward command state duration. For each neuron pair, we first computed raw cross-correlogram counts as follows. We used peaks identified as described in the above section, only during forward command states longer than 50 s. For each peak in the reference neuron (rows in Figures 3C and S2E), all peaks in the target neuron (columns in Figures 3C and S2E) during the same forward command state were considered; in 10 s bins, the time delays of these target neuron peaks relative to the reference peak were accumulated. This procedure was used across all reference peaks within each state, and ultimately to accumulate the full raw cross-correlogram across all forward command states across all recordings. Raw cross-correlogram counts were then converted to frequencies, by dividing by the number of available data for each bin (i.e., the number of possible frames in each bin in which spikes could have occurred, which varies due to differences in trial duration). 10^6^ resampled raw cross-correlograms were computed by randomly selecting the same number of spike times of the target neuron within each forward state and normalizing in the same manner. This resampling procedure accounts for trial-to-trial co-fluctuations in neuronal activity frequencies that may be independent of peak timing relationships but inadvertently cause peaks in the cross-correlogram (Brody, 1999). Covariograms were then computed by subtracting the average resampled raw cross-correlograms (which serves as the “shuffle corrector” or “shift predictor” [Brody, 1999]) from actual raw cross-correlograms. For statistical significance test, see below, section “Quantification and statistical analysis.”

##### Neuronal time series extraction and behavior analysis (freely moving imaging)

GCaMP and mCherry signals acquired at 30 Hz were extracted by first tracking the mCherry signal of the nucleus or cell body of interest using Metamorph software (Molecular Devices). Then, using custom MATLAB (Mathworks) scripts, the coordinates of the tracked regions were used to extract the average of the 50 brightest pixels. A background measurement from the first frame per channel per recording was subtracted. The GCaMP/mCherry ratio, henceforth “R,” was then used to calculate an R_0_ value as the mean of the lowest 10% of ratio values. ΔR/R_0_ was then calculated as (R – R_0_) / R_0_. For subsequent analyses, peaks were detected using a parameter delta determined by manual inspection. A maximum point must be preceded and followed by a value lower by at least delta, and vice versa, a minimum point must be preceded and followed by a value higher by at least delta. Data were further normalized by the 95^th^ percentile in each recording for Figures 5H and S5F.

Reversals were identified manually by examining infrared movies. All other time points were considered as forward states. Custom MATLAB (Mathworks) scripts used a combination of edge detection and gray level thresholding to extract a binary worm image, which were used for subsequent skeletonization as described in (Hums et al., 2016) to quantify 24 angle measurements. Angle measurements were linearly interpolated to match the 30 Hz Ca^2+^ recording and manually inspected for correct ventral/dorsal assignment.

##### Propagation analysis, head-bend type classification, and cycle period quantification (behavior assays and freely moving imaging)

Note that data in Figures 1E, 1F, and S1E–S1G were obtained from Ca^2+^ imaging recordings, as these recordings were high enough resolution (and contained fluoresecent neuronal markers) to distinguish dorsal and ventral sides, and because head-casts were more prominent in these animals allowing us to quantify head-cast episodes containing more than two head-casts. All other data in these figures were obtained in population behavior assays. Kymograms from either population behavior assays or simultaneous Ca^2+^ imaging and behavior experiments were smoothed and, in the case of missing data due to poor skeletonization, linearly interpolated to fill gaps < 1 s. All subsequent analyses were performed separately on forward and reverse locomotion periods. For each angle timeseries, local maxima and minima were detected using a parameter delta determined by manual inspection. A maximum point must be preceded and followed by a value lower by at least delta, and vice versa, a minimum point must be preceded and followed by a value higher by at least delta. Each angle timeseries was thereby transformed into an “angle peak timeseries” containing for each frame either a maximum ( = 1), a minimum ( = −1), or neither ( = 0). Note that maxima (minima) can be dorsal (ventral) if the head-bend changes by at least delta but does not cross 0. The resulting matrix of all angle peak timeseries, or “peak kymogram,” (Figure S1A, lower) of the same size as the original kymogram, was analyzed to determine the propagation of each head-bend as follows.

Because the first (anterior-most) angle is typically noisier than the others, peaks in the second angle were initially defined as head-bends. Head-bends were then analyzed in temporal order, in the following manner. For each head-bend (angle #2 peak), if the previous (i.e., backward or simultaneous in time) peak in angle #1 is of the same sign as the current head-bend (maximum, = 1, or minimum, = −1), and hasn’t been assigned to a previous head-bend, then that peak is assigned to the current head-bend. Similarly, if the next (i.e., forward or simultaneous in time) peak in angle #3 is of the same sign as the current head-bend (maximum, = 1, or minimum, = −1), and hasn’t been assigned to a previous-head-bend, then that peak is assigned to the current head-bend. The next peak in angle #4 is examined with respect to the current peak in angle #3 in the same way; this process is iterated posteriorly until the final angle (see Figure S1A, lower), or until the propagation is terminated as follows. If the next posterior peak is of the opposite sign as the current head-bend, the head-bend is terminated. If the next posterior peak (a) occurs > 10 s following the previous peak and/or (b) occurs following at least two unassigned peaks in the previous (i.e., anterior) angle, then it is likely not part of the current head-bend, so the head-bend propagation is terminated. In both above cases, if the final propagation angle was less than or equal to angle #13, then the head-bend is assigned as a head-cast. If the final propagation angle was angle #14 or greater, then the head-bend is assigned as a propagated-bend. If the animal reverses or if missing data are encountered, the head-bend is terminated as well; in this case, if the final propagation angle is #14 or greater, the head-bend is assigned as a propagated-bend, but if it is less than angle #14, it is ambiguous as to whether the head-bend would have propagated further and is therefore not classified. Head-bends that are not connected to bends in any other angle are also not classified as they are likely due to noise or artifacts in angle measurement. To account for such measurement noise, which may be amplified by peak detection, each head-bend is allowed to propagate backward in time by 0.1 s, and up to 1 s only once per propagation (for example, see Figure S1A, lower, t = 19 s, angle #24). Additionally, two segments per propagation may be skipped if a peak of the correct sign is not detected before the termination condition (for example, see Figure S1A, lower, t = 12 s, angles #22-23). These heuristics enabled the most robust detection of head-bend propagation upon inspection.

Note that for the 2^nd^ head-cast of each pair, there is some ambiguity with regard to whether posterior bends, which are of the same sign, should in fact be assigned to the previous propagated-bend as opposed to that head-cast (in which case that head-cast would instead be assigned as a propagated-bend). Our algorithm assigns these ambiguous posterior bends to the earliest possible head-bend; for example, see Figure S1A, lower, first head-cast pair. Results from SMD and DB02 neuronal activity imaging during behavior suggest that this procedure meaningfully assigns different head-bend types, because neuronal activity during the earlier head-bend is indistinguishable from other propagated-bends, whereas neuronal activity during the later head-bend is markedly different (Figures 5C and 7E).

Reversal states were analyzed the same way, only with kymograms flipped left/right such that bends are quantified as if they propagated head-to-tail, as during forward locomotion, rather than tail-to-head. This allows the classification of bends propagating fully from tail to head as well as bends that start in the neck and propagate toward the head, which for Figure S1J were considered head-casts. Also considered head-casts during reversals for Figure S1J were bends that propagate as head-casts normally do, which were found by analyzing reversal states without flipping left/right.

Because head-casts typically occurred in only half-cycles (i.e., two head-casts, one ventral and one dorsal, without a return to the side of the first head-cast), head-cast cycle period in Figure 1D was calculated by doubling head-cast inter-bend intervals (only measured between two consecutive head-casts). For Figure 1D propagated-bend cycle periods were calculated regardless of intervening head-cast interruption; note that a reduction in head-casts caused only a small change in propagated-bend inter-bend intervals (Figure S4D). Note that head-casts were typically posterior to and slower than the 4-5Hz nose-tip movements previously described as “foraging,” (Huang et al., 2008) and were more in line with head movements described in refs. (McCloskey et al., 2017, Skora et al., 2018). Still, in agreement with (Yemini et al., 2013), we believe that previously described foraging movements include head-casts, yet we use a different term to distinguish our definition based on propagation.

For Figure 8, propagated-bend cycle periods were calculated by doubling propagated-bend inter-bend intervals. For Figures 8G and 8H, data are taken from 10min post-lights-on. For Figure 5I, head-bend amplitude is the summed amplitudes of segment numbers 2 to 6.

##### Phase quantifications

Phases in Figures 1F, 7G, and 7H were quantified by linearly interpolating between detected peaks for each half cycle (a similar procedure was used by (Eliav et al., 2018)). Interpolation enabled a phase calculation that was independent of intervening head-casts or non-alternating SMD oscillations. In Figure 1F, 0 to π was interpolated from dorsal to ventral propagated-bend maxima (peaks in head-bend angle #2) and π to 2π was interpolated from ventral to dorsal propagated-bend maxima, regardless of any intervening head-casts. Ventral and dorsal head-casts were defined as head-casts following ventral or dorsal propagated-bends, respectively; each head-cast type was therefore restricted to a half cycle. Only the first head-cast following a propagated-bend was used to generate the histograms in Figure 1F. In Figures 7G and 7H, 0 to π was interpolated from the first SMDD peak following an SMDV peak (i.e., SMDD alternating peak) to the first SMDV peak following an SMDD peak, regardless of any intervening SMDD peaks. Similarly, π to 2π was interpolated from the first SMDV peak following an SMDD peak to the first SMDD peak following an SMDV peak, regardless of any intervening SMDV peaks. Because of this definition, SMDD-only and SMDV-only oscillations were each restricted to half cycles. To best compare to Figure 1F, we used the first trough (i.e., minimum, see Figure 7E, t = ∼0 s) of each SMDD-only or SMDV-only oscillation to generate the histograms, as these corresponded to the first head-cast following a propagated-bend.

Phases in Figure 5D–5G were calculated using the Hilbert transform of head-bend angle #4, smoothed, to remove head-cast signals. The Hilbert transform was applied individually to each period of forward or reverse locomotion, and border data were removed as these were often partial cycles. To quantify the proportion of cycles that contained neural activity peaks, individual cycles were segmented from 0 to 2π (SMDV) or from π to π (SMDD and DB02) so that the typical peak timing for each neuron was centered and was therefore unlikely to fall into a neighboring cycle due to noise. Only cycles without missing data in both angle and neural activity were considered.

### Quantification and Statistical Analysis

Quantifications were performed using custom MATLAB (Mathworks) scripts. Standard statistical tests were performed using Graphpad Prism 7. These tests, along with the value of n and what n represents, are reported in the figure legends. Additional tests are described in detail in the following sections.

#### Reconstruction quality for neuronal subsets (pan-neuronal imaging)

To assess how well interneuron and motor neuron activity was captured in the top PC dimensions (Figure 2E; only identified neurons used), we calculated for each neuron the Pearson’s linear correlation coefficient between the original data (activity traces and their derivatives) and reconstructed traces after PCA. These reconstructed traces were obtained by matrix multiplication of the PC coefficients with the PC loadings of the top (i = 1 to 5) PCs and addition of the mean of the original data, as follows:

$$Data_{recon}=\left[PC_{Coeff^{1}}:PC_{Coeff^{i}}\right]\times \left[PC_{Load^{1}}:PC_{Load^{i}}\right]+mean\left(OrigData\right).$$

We used the average correlation coefficient for all the traces of a neuronal cell type (inter neurons, motor neurons or other neurons) in a recording as a measure of how similar reconstructed traces are to their original counterparts. This measure, in contrast to mean-square-error, is insensitive to the magnitude of neuronal activity traces.

#### Peak frequency quantification (pan-neuronal imaging)

For all neurons that were identified in at least three recordings, we tested whether the inter-peak interval distribution between such peaks, across all such forward command states, was significantly different from random, as follows. For each forward command state, we randomly selected the same number of spike times to accumulate a random inter-peak interval distribution. This procedure was repeated 10^6^ times to obtain an average random distribution. To measure the magnitude of deviation from random, we summed the absolute difference, bin-by-bin, between the average random distribution and the actual inter-peak interval distribution. This measure was also determined for each of the 10^6^ resampled distributions, and the p value reported in Table S2 is the fraction of resampled distributions with at least as large of a deviation from random as the actual distribution. To correct for multiple comparisons, statistical significance was determined by the Benjamini-Hochberg-Yekutieli procedure (Benjamini and Yekutieli, 2001).

#### Cross-correlation analysis (pan-neuronal imaging)

Statistical significance was evaluated by comparing the actual and resampled covariograms as follows. First, the covariogram was assigned a positive or negative relationship, depending on whether the absolute value of the maximum or the minimum value of the covariogram was larger (across all time bins); negative relationships found in this manner are marked with “(-)” in Figure 3C. Then, each of the 10^6^ resampled distributions were analyzed to determine the probability of finding correlations with absolute values as large as either the maximum (for previously determined positive relationships) or minimum (for negative relationships) value observed in the actual covariogram. The p value reported in Table S3 is the fraction of maxima or minima that are at least as large in absolute value as that obtained from the actual distribution. To correct for multiple comparisons across all neuron pairs, statistical significance was determined by the Benjamini-Hochberg-Yekutieli procedure (Benjamini and Yekutieli, 2001)

#### Polar histogram statistics

We tested the significance of the distributions in Figures 1F, 5D–5G, 7G, and 7H using resampling. We determined the probability of obtaining a distribution as skewed as the real one by chance. For each half-cycle (Figures 1F, 7G, and 7H) or full cycle (Figures 5D–5G) from which the real data had been extracted, we randomly selected one phase, to generate a random distribution of phases of the same number as the real distribution. Because peaks were detected using a parameter delta for Figures 1F, 7G, and 7H, we restricted the time points that could be selected to those where peaks could possibly have been found (i.e., those at least delta away from the previous peak). We binned the data in the same manner as the real data and quantified the absolute difference, bin-by-bin, between the resampled distribution and the all-phases distribution, which was calculated using the same phases from which the random data was selected. These bin-by-bin absolute differences were summed to give a total difference between the resampled data and the all-phases distribution as a measure of sample distribution skewness. This procedure was repeated 10^6^ times and the skewness of the real distribution was measured the same way. The p value is the fraction of randomly sampled distributions that were at least as skewed as the actual distribution.

Significance between forward (Figure 5F) and reverse (Figure 5G) SMD peak distributions were determined by resampling independently for each SMD. The reverse distribution contained fewer peaks than the forward distribution, so we randomly selected, from the forward distribution, 10^6^ samples of the same size as the reverse distribution. We binned the data in the same manner as the real data and quantified, bin-by-bin, the absolute difference between the resampled and real forward distribution. These bin-by-bin absolute differences were summed to give a total difference. The same procedure was done to measure the difference between the forward and reversal distributions, and the p value is the fraction of randomly sampled forward distributions that differed from the actual forward distribution at least as much as the reversal distribution did.

### Data and Code Availability

Data and custom code generated in this study are available from the Lead Contact without restriction.

## Supporting Citations

The following references appear in the Supplemental Information: Bhatla and Horvitz, 2015, Ringstad et al., 2009, Ruijtenberg and van den Heuvel, 2015, Sohal et al., 2009.

## Supplemental Information

Document S1. Figures S1–S8 and Tables S1–S3

Document S2. Article plus Supplemental Information
