# Supplementary material for: Nested Neuronal Dynamics Orchestrate a Behavioral Hierarchy across Timescales
Source: Neuron. 2020 Feb 5;105(3):562–576.e9. doi: 10.1016/j.neuron.2019.10.037 (PMC7014571; doi:10.1016/j.neuron.2019.10.037)
Supplement: Document S1. Figures S1–S8 and Tables S1–S3 [file mmc1.pdf]

Neuron, Volume *105*

Supplemental Information

Nested Neuronal Dynamics Orchestrate a Behavioral Hierarchy across Timescales

Harris S. Kaplan, Oriana Salazar Thula, Niklas Khoss, and Manuel Zimmer

Figure S1

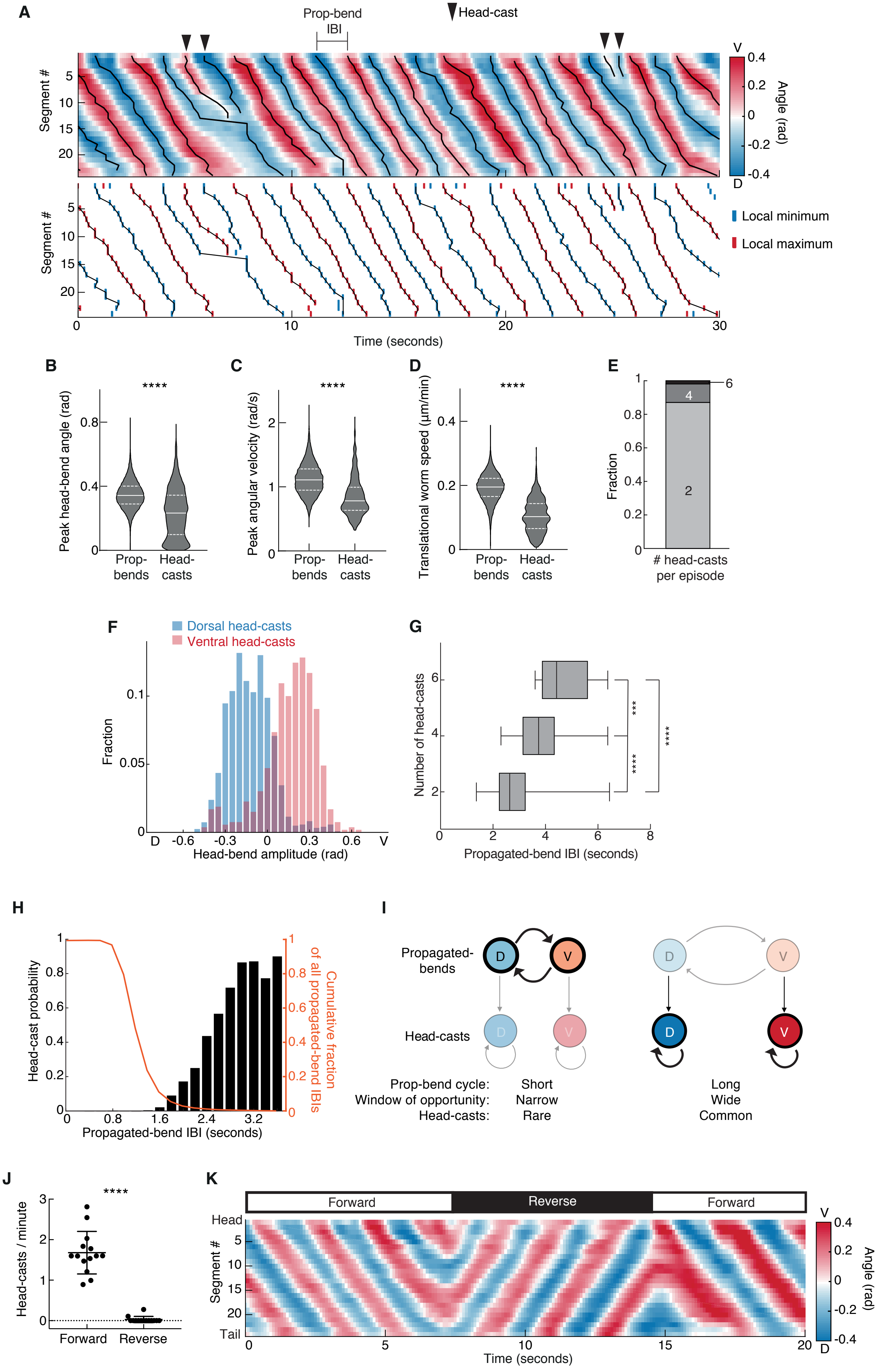
A
Prop-bend
IBI
Head-cast
Segment #
5
10
15
20
V
0.4
0.2
0
-0.2
-0.4
D
Angle (rad)
Local minimum
Local maximum
0
10
20
30
Time (seconds)
B
****
Peak head-bend angle (rad)
0.8
0.4
0
Prop-bends
Head-casts
C
****
Peak angular velocity (rad/s)
2
1
0
Prop-bends
Head-casts
D
****
Translational worm speed (µm/min)
0.4
0.2
0
Prop-bends
Head-casts
E
Fraction
1
0.8
0.6
0.4
0.2
0
6
4
2
head-casts per episode
F
Dorsal head-casts
Ventral head-casts
Fraction
0.1
0.05
0
D
-0.6
-0.3
0
0.3
0.6
V
Head-bend amplitude (rad)
G
Number of head-casts
6
4
2
0
2
4
6
8
***
****
****
Propagated-bend IBI (seconds)
H
Head-cast probability
1
0.8
0.6
0.4
0.2
0
0
0.8
1.6
2.4
3.2
Propagated-bend IBI (seconds)
Cumulative fraction of all propagated-bend IBIs
I
Propagated-bends
Head-casts
D
V
Prop-bend cycle:
Window of opportunity:
Head-casts:
Short
Narrow
Rare
Long
Wide
Common
J
****
Head-casts / minute
3
2
1
0
Forward
Reverse
K
Forward
Reverse
Forward
Head
Tail
Segment #
5
10
15
20
0
5
10
15
20
Time (seconds)
V
0.4
0.2
0
-0.2
-0.4
D
Angle (rad)

Figure S2

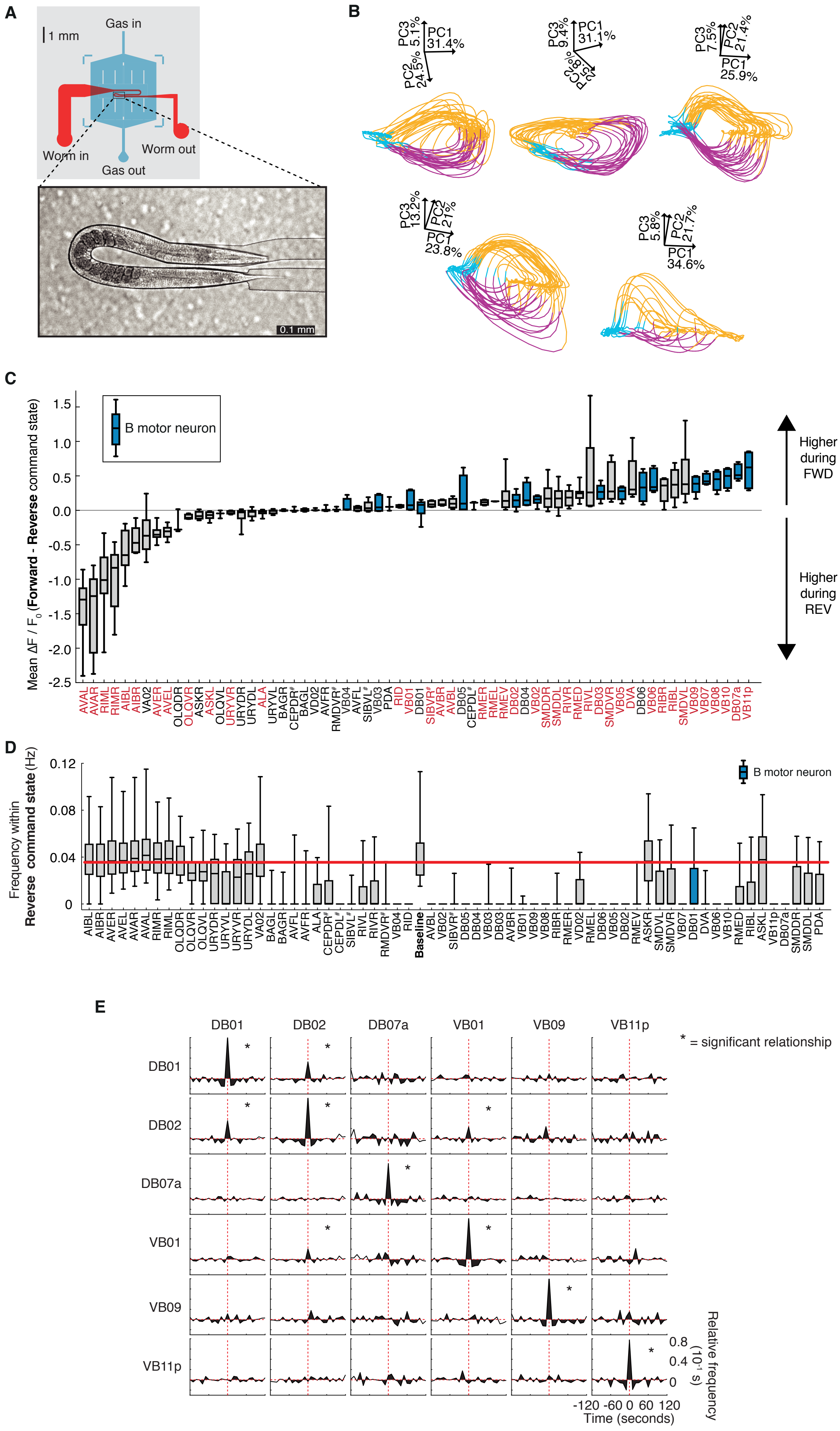

A
1 mm
Gas in
Worm in
Worm out
Gas out
0.1 mm
B
PC1 31.4%
PC2 24.5%
PC3 5.1%
C
B motor neuron
Higher during FWD
Higher during REV
Mean ΔF / F0 (Forward - Reverse command state)
D
B motor neuron
Frequency within Reverse command state (Hz)
Baseline
E
DB01
DB02
DB07a
VB01
VB09
VB11p
* = significant relationship
Relative frequency (10⁻¹ s)
Time (seconds)

Figure S3

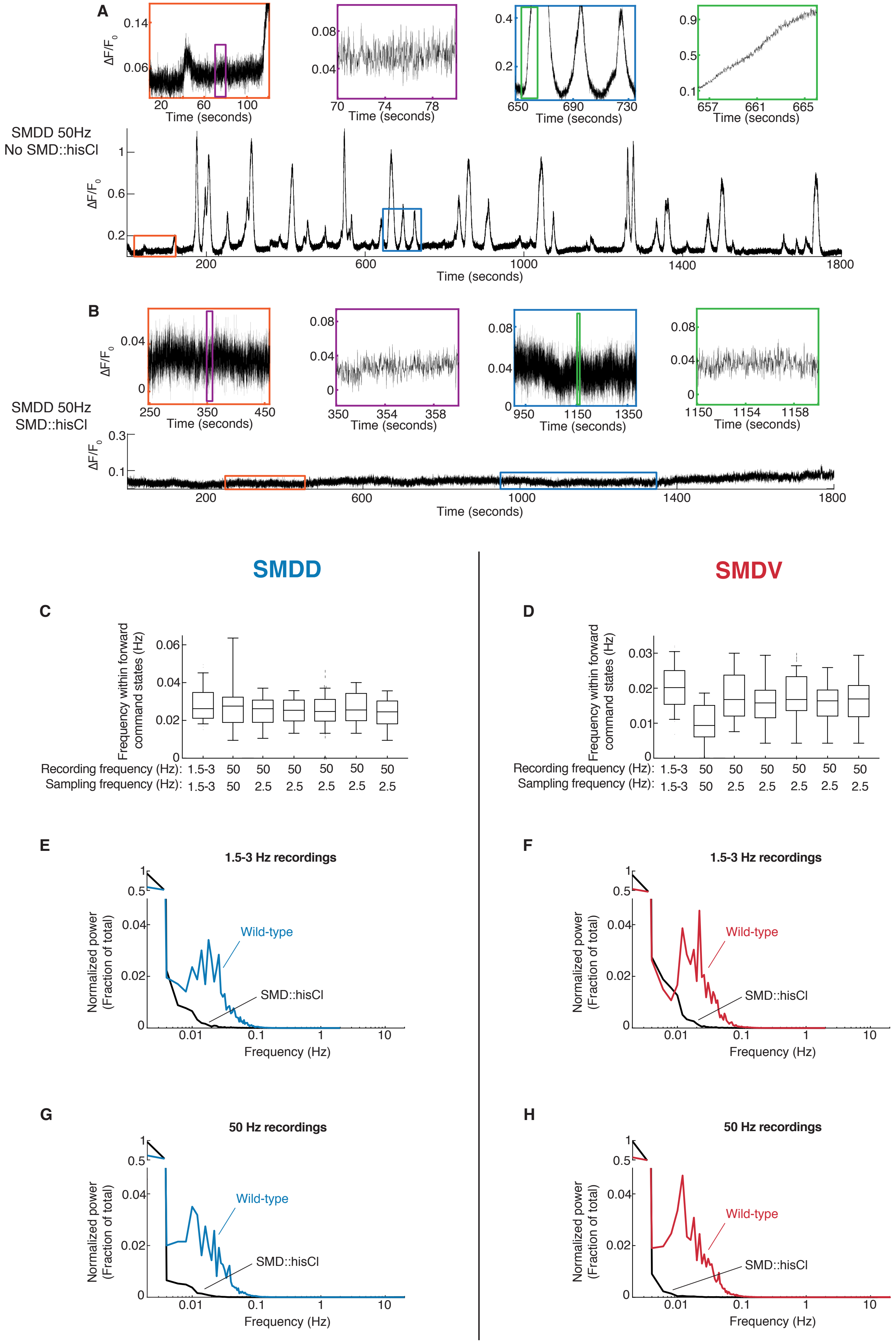

A
ΔF/F0
0.14
0.06
20
60
100
Time (seconds)
0.08
0.04
70
74
78
Time (seconds)
0.4
0.2
650
690
730
Time (seconds)
0.9
0.5
0.1
657
661
665
Time (seconds)
SMDD 50Hz
No SMD::hisCl
1
0.6
0.2
200
600
1000
1400
1800
Time (seconds)
B
0.04
0
250
350
450
Time (seconds)
0.08
0.04
0
350
354
358
Time (seconds)
0.08
0.04
0
950
1150
1350
Time (seconds)
0.08
0.04
0
1150
1154
1158
Time (seconds)
SMDD 50Hz
SMD::hisCl
0.3
0.1
200
600
1000
1400
1800
Time (seconds)
SMDD
SMDV
C
Frequency within forward command states (Hz)
0.06
0.04
0.02
0
Recording frequency (Hz): 1.5-3 50 50 50 50 50 50
Sampling frequency (Hz): 1.5-3 50 2.5 2.5 2.5 2.5 2.5
D
Frequency within forward command states (Hz)
0.03
0.02
0.01
0
Recording frequency (Hz): 1.5-3 50 50 50 50 50 50
Sampling frequency (Hz): 1.5-3 50 2.5 2.5 2.5 2.5 2.5
E
1.5-3 Hz recordings
Normalized power (Fraction of total)
1
0.5
0.04
0.02
0
Wild-type
SMD::hisCl
0.01
0.1
1
10
Frequency (Hz)
F
1.5-3 Hz recordings
Normalized power (Fraction of total)
Wild-type
SMD::hisCl
Frequency (Hz)
G
50 Hz recordings
Normalized power (Fraction of total)
Wild-type
SMD::hisCl
Frequency (Hz)
H
50 Hz recordings
Normalized power (Fraction of total)
Wild-type
SMD::hisCl
Frequency (Hz)

Figure S4

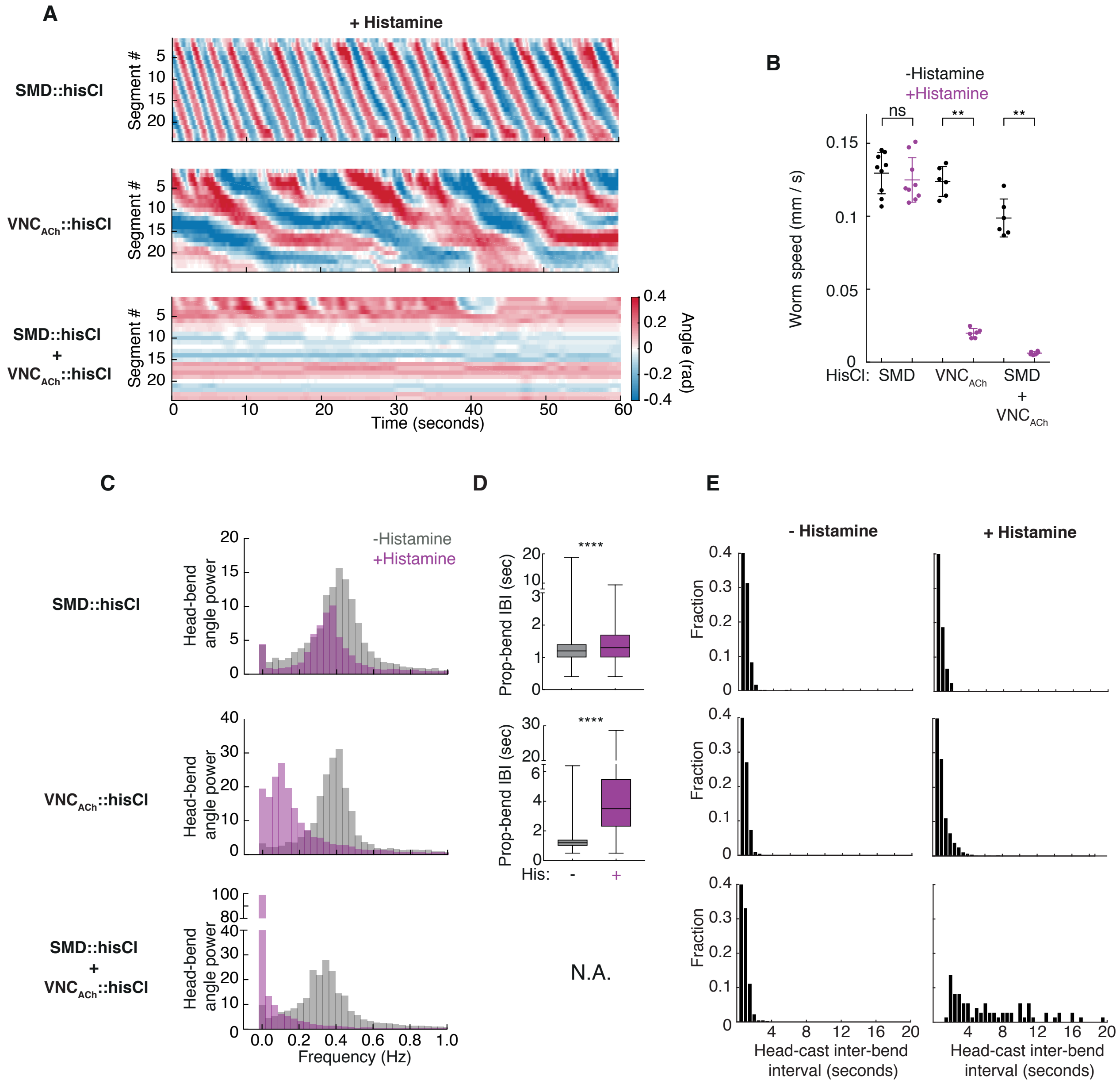
A
+ Histamine
SMD::hisCl
VNC$_{ACh}$::hisCl
SMD::hisCl
+
VNC$_{ACh}$::hisCl
Segment #
5
10
15
20
0.4
0.2
0
-0.2
-0.4
Angle (rad)
0
10
20
30
40
50
60
Time (seconds)
B
-Histamine
+Histamine
ns
**
**
Worm speed (mm / s)
0.15
0.1
0.05
0
HisCl: SMD
VNC$_{ACh}$
SMD
+
VNC$_{ACh}$
C
-Histamine
+Histamine
SMD::hisCl
VNC$_{ACh}$::hisCl
SMD::hisCl
+
VNC$_{ACh}$::hisCl
Head-bend angle power
Frequency (Hz)
0.0
0.2
0.4
0.6
0.8
1.0
D
Prop-bend IBI (sec)
****
****
His: -
+
N.A.
E
- Histamine
+ Histamine
Fraction
Head-cast inter-bend interval (seconds)
4
8
12
16
20

Figure S5

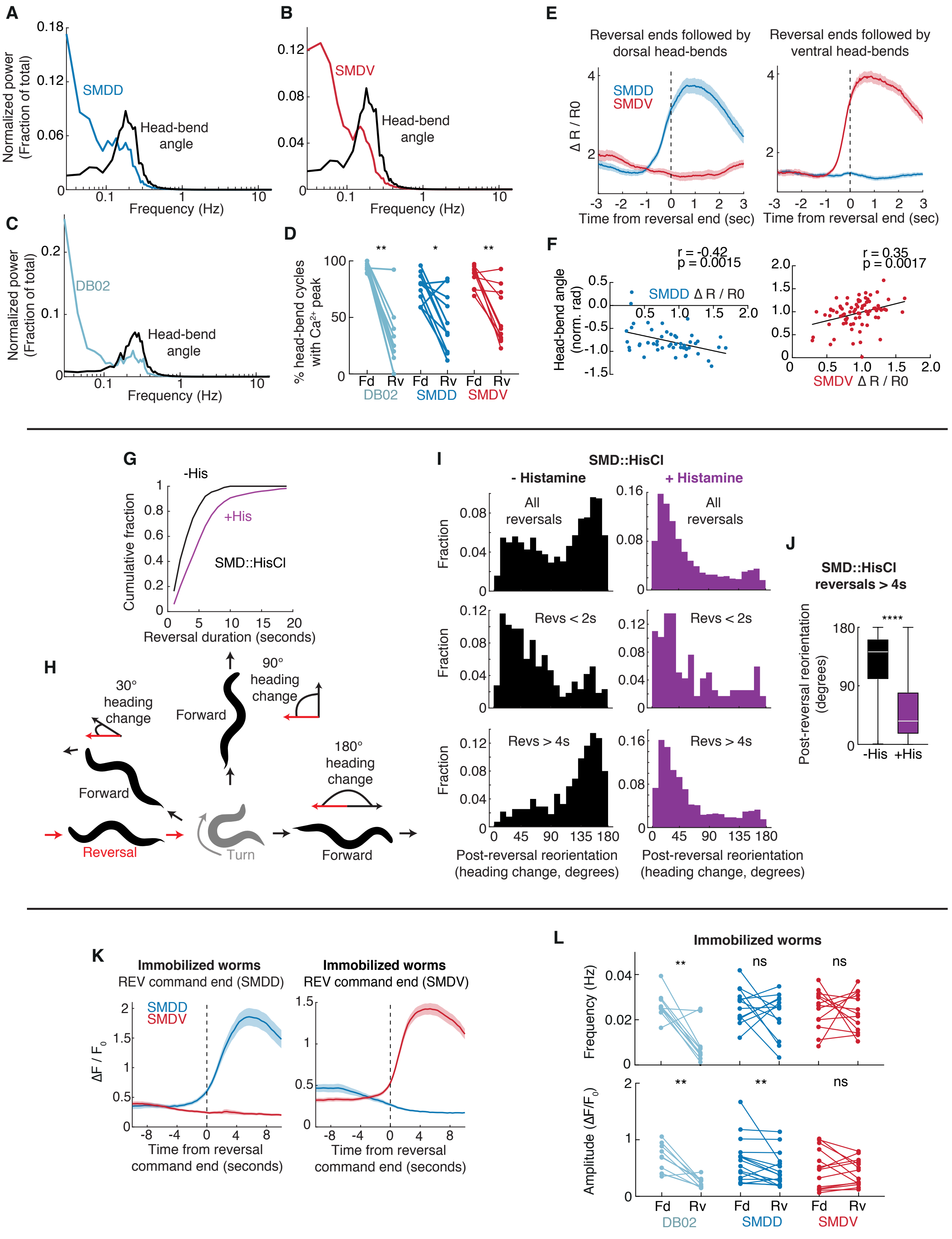
A
Normalized power (Fraction of total)
SMDD
Head-bend angle
Frequency (Hz)
B
SMDV
Head-bend angle
Frequency (Hz)
C
Normalized power (Fraction of total)
DB02
Head-bend angle
Frequency (Hz)
D
% head-bend cycles with Ca2+ peak
Fd Rv Fd Rv Fd Rv
DB02 SMDD SMDV
E
Reversal ends followed by dorsal head-bends
Reversal ends followed by ventral head-bends
Δ R / R0
SMDD
SMDV
Time from reversal end (sec)
F
Head-bend angle (norm. rad)
r = -0.42
p = 0.0015
SMDD Δ R / R0
r = 0.35
p = 0.0017
SMDV Δ R / R0
G
-His
+His
SMD::HisCl
Cumulative fraction
Reversal duration (seconds)
H
30° heading change
90° heading change
180° heading change
Forward
Reversal
Turn
I
SMD::HisCl
- Histamine
+ Histamine
All reversals
Revs < 2s
Revs > 4s
Fraction
Post-reversal reorientation (heading change, degrees)
J
SMD::HisCl reversals > 4s
Post-reversal reorientation (degrees)
****
-His +His
K
Immobilized worms
REV command end (SMDD)
REV command end (SMDV)
SMDD
SMDV
ΔF / F0
Time from reversal command end (seconds)
L
Immobilized worms
Frequency (Hz)
Amplitude (ΔF/F0)
** ns ns
** ** ns
Fd Rv Fd Rv Fd Rv
DB02 SMDD SMDV

Figure S6

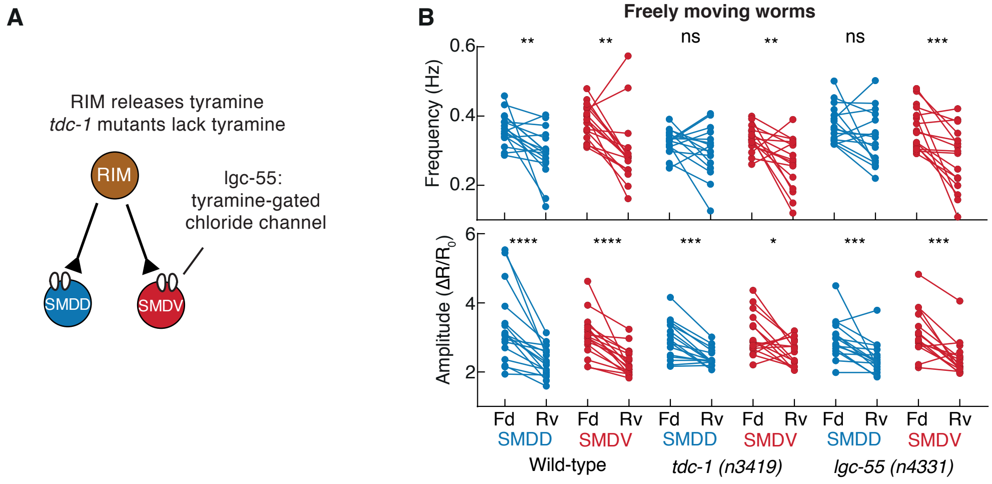
A
RIM releases tyramine
tdc-1 mutants lack tyramine
RIM
lgc-55:
tyramine-gated
chloride channel
SMDD
SMDV
B
Freely moving worms
Frequency (Hz)
0.6
0.4
0.2
** ** ns ** ns ***
Amplitude (ΔR/R0)
6
4
2
**** **** *** * *** ***
Fd Rv Fd Rv Fd Rv Fd Rv Fd Rv Fd Rv
SMDD SMDV SMDD SMDV SMDD SMDV
Wild-type
tdc-1 (n3419)
lgc-55 (n4331)

Figure S7

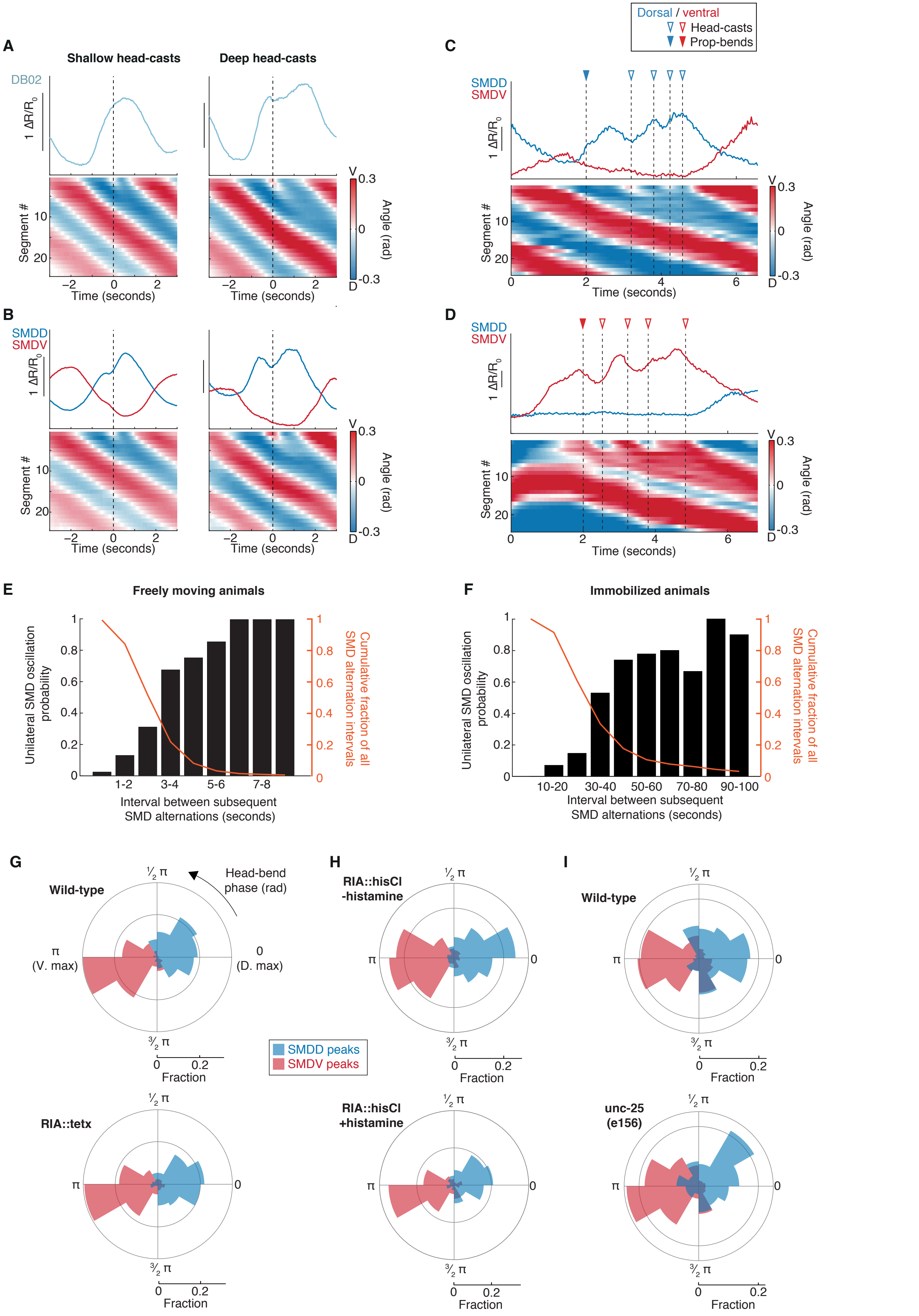
A
Shallow head-casts
Deep head-casts
DB02
1 ΔR/R0
Segment #
Time (seconds)
Angle (rad)
V
D
0.3
0
-0.3
B
SMDD
SMDV
C
Dorsal / ventral
Head-casts
Prop-bends
D
E
Freely moving animals
Unilateral SMD oscillation probability
Cumulative fraction of all SMD alternation intervals
Interval between subsequent SMD alternations (seconds)
1-2
3-4
5-6
7-8
F
Immobilized animals
10-20
30-40
50-60
70-80
90-100
G
Wild-type
Head-bend phase (rad)
π (V. max)
0 (D. max)
½ π
3/2 π
Fraction
RIA::tetx
SMDD peaks
SMDV peaks
H
RIA::hisCl -histamine
RIA::hisCl +histamine
I
unc-25 (e156)

Figure S8

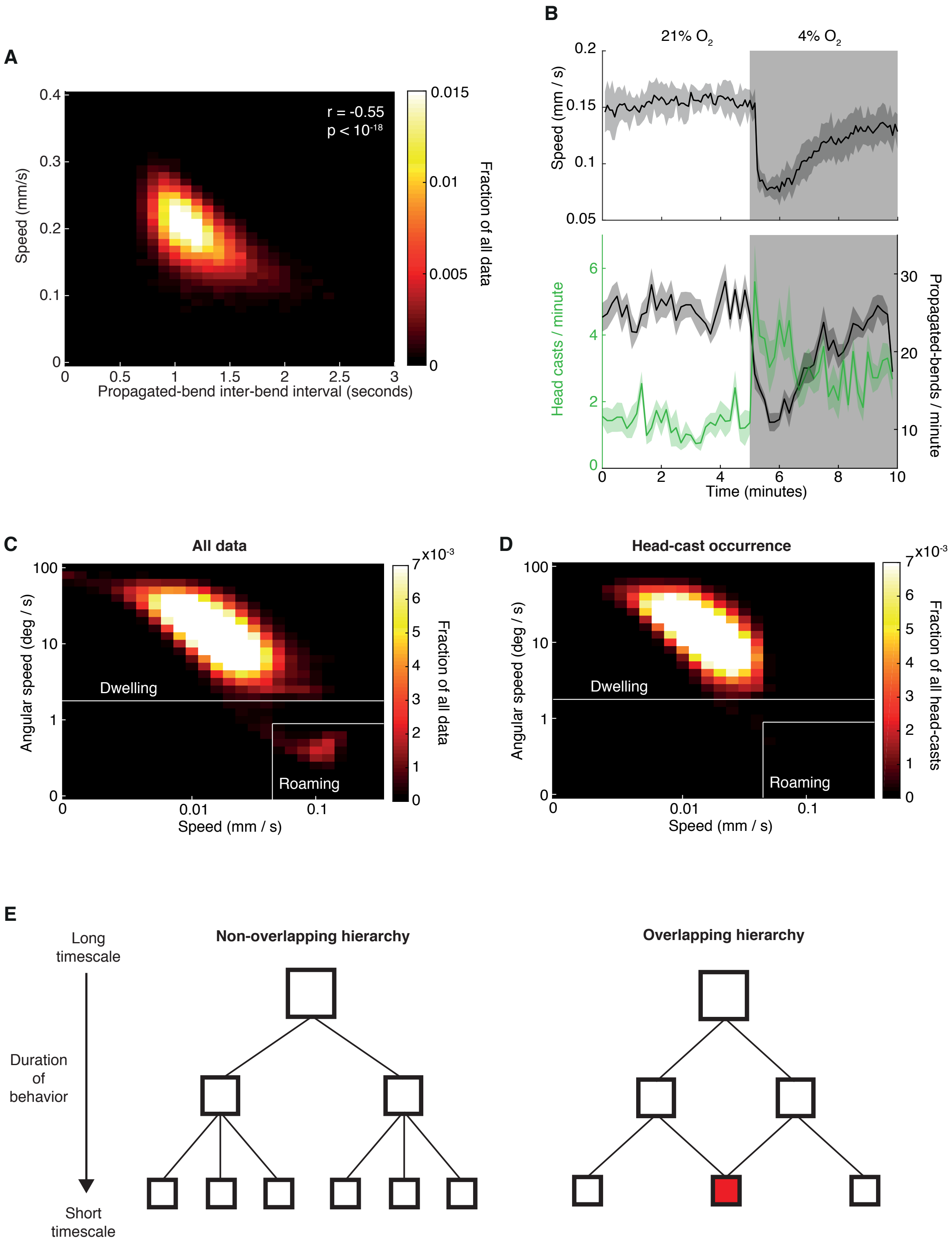
A
r = -0.55
p < 10-18
Speed (mm/s)
Propagated-bend inter-bend interval (seconds)
Fraction of all data
B
21% O2
4% O2
Speed (mm / s)
Head casts / minute
Propagated-bends / minute
Time (minutes)
C
All data
Angular speed (deg / s)
Dwelling
Roaming
Speed (mm / s)
Fraction of all data
D
Head-cast occurrence
Angular speed (deg / s)
Dwelling
Roaming
Speed (mm / s)
Fraction of all head-casts
E
Long timescale
Duration of behavior
Short timescale
Non-overlapping hierarchy
Overlapping hierarchy

Supplementary Figure Legends.

Figure S1. Kinematic relationships between different timescale behaviors, related to Figure 1. (A) Upper: example kymogram showing a period of forward movement. Head-bend propagation traces shown by black lines. Arrowheads mark head-casts. Lower: peak kymogram showing detected maxima and minima used for propagation tracing (Methods). **(B-D)** Violin plots showing median and 1st and 3rd quartiles of (**B**) absolute peak head-bend angle (segment #2) during propagated-bends (n=44487) or head-casts (n=2987); **(C)** absolute peak angular velocity of the head (segment #2) during propagated-bend half-oscillations (n=25397) or head-cast half-oscillations (n=529); and **(D)** average translational worm speed during propagated-bend half-oscillations (n=25397) or head-cast half-oscillations (n=529). ****p<0.0001, Mann-Whitney Test. Data in (**B-D**) are pooled from 14 assays, ~20 animals per assay. **(E)** Number of head-casts per head-cast episode. A head-cast episode is defined as a series of consecutive head-casts uninterrupted by propagated-bends or reversals (i.e. within one propagated-bend inter-bend interval (IBI), illustrated in **(A)**). n = 604 head-casts pooled from 21 animals. **(F)** Fractional histograms of maximal head-bend amplitude (segment #2) for dorsal or ventral head-casts, defined according to whether they followed dorsal or ventral propagated-bends, respectively. n = 427 dorsal and 194 ventral head-casts pooled from 21 animals. Note that head-casts can cross to the contralateral side of the previous propagated bend, but are largely restricted to the ipsilateral side. **(G)** Median, interquartile and range of propagated-bend inter-bend intervals (IBI, illustrated in **(A)**) for propagated-bend pairs with either 2, 4 or 6 intervening head casts. n = 525 (2 head-casts), 67 (4 head-casts) and 12 (6 head-casts) episodes

pooled from 14 experimental repeats with ~20 animals each. ***$p<0.001$, ****$p<0.0001$, Mann-Whitney Test. **(H)** Probability of at least one head-cast occurrence between a pair of propagated-bends, binned according to the propagated-bend pair's IBI (as illustrated in (**A**). Cumulative fraction of all propagated-bend IBIs overlaid in orange: note that head-casts are most probable during the longest propagated-bend cycles. n=25815 propagated-bend pairs and 420 head-casts pooled from 14 assays, ~20 animals per assay. **(I)** Diagram illustrating how the propagated-bend cycle could act as a window of opportunity for head-casting, which may be widened or narrowed by decreasing or increasing cycle speed. **(J)** Head-cast frequency (regardless of propagation direction) in forward vs. reverse locomotion. Each data point is the mean of an experimental replicate, n = 14 with ~20 animals per replicate. ****$p<0.0001$, Mann-Whitney Test. **(K)** Example kymogram showing an animal switching between forward and reverse locomotion states. Note that during reverse locomotion, body undulations are initiated in the tail and propagate to the head, in contrast to forward locomotion body undulations which are propagated posteriorly. We use the term "propagated-bends" throughout to refer only to the latter.

Figure S2. Nervous-system-wide Ca^{2+} imaging reveals motor neuron activity nested within forward/reverse command switches, related to Figures 2 and 3. **(A)** Technical drawing of microfluidic device used for whole-brain and whole-nervous-system Ca^{2+} imaging experiments. Black box shows a zoom-in of an example worm immobilized in the imaging curve. **(B)** PC-phase plots for each of five nervous-system-wide recordings, as in **Fig. 2C**. Traces colored by instantaneous motor command state inferred from neuronal activity (Methods); color key in **Fig.**

2C, lower panel (arrowheads indicate directional flow). Coordinates depict PC axes orientations and % variance explained. Compare to Ref. 8 showing head ganglia PC-phase plots. **(C)** Difference in mean Ca^{2+} activity levels (ΔF / F0) of all identified neurons during forward vs. reverse commands, pooled from n = 5 whole-nervous-system and n= 5 whole-brain recordings. Boxes: median and interquartile; whiskers: range. Blue boxes correspond to B motor neurons. Neurons labeled in red are those with significantly different mean activity levels in forward vs. reverse commands (paired t-test); see **Table S2** for p-values and n numbers. #ambiguous IDs, see Methods for alternatives. **(D)** Frequencies of activity peaks of all identified neurons within reverse command states, with one frequency data point calculated for each reverse command state, pooled from n=5 whole-nervous-system and n=5 whole-brain recordings. Boxplots show median and interquartile; whiskers show 5%-95% range. "Baseline" is the distribution for one peak per reversal command state; its median is indicated by the red line. Neurons ordered according to median frequency in forward state, see **Fig. 3A**. Note that more neurons appear active in the forward state because we only include neuron classes which we reliably identified; we focused on identifying forward-active B-MNs and not reverse-active A-MNs, although we observed strong signals in the latter (data not shown). **(E)** Covariograms of oscillatory motor neurons. Panels show the relative frequencies of Ca^{2+}-peaks of neurons in columns triggered to Ca^{2+}-peaks of neurons in rows. Data are shuffle-corrected so that positive/negative values show higher/lower correlation than chance (Methods). * significant relationship, after multiple comparison correction. All significant relationships are positive. See **Table S3** for p-values.

Figure S3. SMD neuronal activity in immobilized animals is low frequency regardless of sampling rate. Related to Figure 3. (A-B) Example SMDD activity traces from 50 Hz single-plane recordings and zoom-ins without (**A**) or with (**B**) SMD::hisCl, which is a transgene that expresses the histamine-gated chloride channel hisCl specifically in the SMD neurons (all recordings made after histamine incubation; *C. elegans* neither produces nor senses histamine endogenously). Note that the high-frequency signals in SMD::hisCl (purple and green boxes) resemble those in WT animals (purple box), indicating that these changes are likely due to measurement noise rather than calcium changes. **(C-D)** Median, interquartile range and 10-90% interval showing detected activity peak frequency within forward command states (as in Figure 3A) for SMDD (**C**) and SMDV (**D**), in 1.5-3 Hz multi-plane, brain-wide recordings (first column), in 50 Hz single-plane recordings (second column), and in 50 Hz recordings that were down-sampled to 2.5 Hz (last five columns). Note that neither recording rate nor sampling rate affect the number of detected peaks. N numbers for (**C**): 30 states from 5 recordings (column 1), 42 states from 6 recordings (columns 2-7). N numbers for (**D**): 30 states from 5 recordings (column 1), 35 states from 4 recordings (columns 2-7). (**E-H**) Normalized power spectra of SMDD (**E, G**) or SMDV (**F, H**) activity in wild-type (colored traces) or SMD::hisCl (black traces) animals, recorded at 1.5-3 Hz (**E-F**) or 50 Hz (**G-H**). Note that recording at 50 Hz does not reveal any contribution from frequencies higher than those captured at 1.5-3 Hz. N = 5 recordings for each condition except SMDD in WT animals at 50 Hz (n = 6) and SMDV in SMD::hisCl animals at 50 Hz (n = 4).

Figure S4. Behavioral effects of neuronal inhibition, related to Figure 4. (A) Example kymograms from SMD::hisCl (upper), VNC_{ACh}::hisCl (middle) and SMD::hisCl + VNC_{ACh}::hisCl (lower) animals. Note that any coordination among body segments is lost in SMD::hisCl + VNC_{ACh}::hisCl animals. **(B)** Effects of indicated hisCl transgenes on locomotion speed. Bars show mean ± SD. Each data point is the mean of an experimental repeat with ~20 animals each. ns, not significant; **p<0.01, Mann-Whitney Test. **(C-E)** Power spectra histograms of head-bend angle (angle #2) (**C**), propagated-bend inter-bend interval (**IBI**; **D**), and fractional histograms of head-cast IBI **(E)** in SMD::hisCl (upper), VNC_{ACh}::hisCl (middle), and SMD::hisCl + VNC_{ACh}::hisCl (lower) animals with or without histamine. Note that SMD inhibition slightly but significantly increased, rather than decreased, propagated-bend inter-bend interval, indicating that the head-cast reduction in SMD::hisCl did not result in faster propagated-bend cycles (i.e. head-cast occurrences do not stall the propagated-bend cycle, a possibility suggested by data in **Fig. S1H**). SMD::hisCl: n = 11952 (**D**, -his), 11230 (**D**, +his), 1481 (**E**, -his), 211 (**E**, +his). VNC_{ACh}::hisCl: n = 10511 (**D**, -his), 1062 (**D**, +his), 274 (**E**, -his), 1159 (**E**, +his). SMD::hisCl + VNC_{ACh}::hisCl: n = 492 (**E**, -his) and 73 (**E**, +his). Data are averaged **(B)** or pooled **(C-E)** across n = 6 (VNC_{ACh}::hisCl and SMD::hisCl + VNC_{ACh}::hisCl) or n = 9 (SMD::hisCl) experimental repeats with ~20 animals each. ****p<0.0001, Mann-Whitney test.

Figure S5. Interactions between global forward/reverse cycle and SMD/DB oscillators, related to Figure 5. (A-C) Normalized power spectra of SMDD (**A**), SMDV (**B**), and DB02 (**C**) activity, overlaid with the power spectra of the co-recorded head-bend angle in black. The leftmost bin (0 to 0.02 Hz) is removed, as the

spectrum is otherwise dominated by the effects of long-timescale changes in reversal behavior on SMD activity (see **Fig. 5H**). Note the overlap of activity and head-bend angle distributions. Compare to power spectra in immobilized animals (**Fig. S3E-H**) – SMD activity in immobilized worms is about one order of magnitude slower. We suspect the same is true for DB02 given our peak detection analysis (**Fig. 3A**), however we did not record DB02 at high temporal resolution. **(D)** Percentage of all head-bend cycles with at least one activity peak during forward or reverse locomotion. Each data point is from one animal, n=11 (SMD) and n=10 (DB02). *p<0.05, **p<0.01, Wilcoxon matched-pairs signed rank test. **(E)** Trigger-averaged GCaMP/mCherry traces ± SEM from SMD recordings in freely moving animals aligned to reverse-to-turn transitions, separated by post-reversal dorsal (left) or ventral (right) head-bend. n = 55 dorsal and 96 ventral events, pooled from 11 animals. **(F)** Peak head-bend angle (angle #2, normalized to 95th percentile within each recording) and GCaMP/mCherry signal amplitude for SMDD and post-reversal dorsal head-bends (left) or SMDV and post-reversal ventral head-bends (right). Pearson correlation coefficient and p-value indicated, n as in **(E)**. **(G)** Cumulative distribution of reversal duration in SMD::hisCl animals ±histamine. n = 1416 (control) and 1814 (histamine) reversals. p<0.0001, two-sample Kolmogorov-Smirnov test. Note that SMD inhibition led to reversal lengths beyond 10s that were never observed in non-inhibited controls. **(H)** Illustration of different heading changes upon reversal end as quantified in (**I-J**). **(I)** Fractional histograms of absolute heading change (quantified as shown in **(H)**) resulting from reversals of the indicated durations, in SMD::hisCl animals ±histamine. Reversals > 4s were typically followed by strong reorientations, which were lost in SMD::hisCl animals. Control: n = 821 (all), 215 (<2s), and 275 (>4s) reversals; histamine: n = 1230 (all),

118 (<2s), and 848 (>4s) reversals. **(J)** Median, interquartile and range of heading change in SMD::hisCl animals for reversals >4s with or without histamine. n as in (**I**). ****p<0.0001, Mann-Whitney test. Data in (**G-J**) pooled from n = 9 experimental repeats from each condition, with ~20 animals each repetition. **(K)** Trigger-averaged SMD GCaMP traces ± SEM from immobilized worm recordings aligned to reverse-to-forward command state transitions, separated by SMDD (left) and SMDV (right) transition peaks. n = 64 (SMDD) and 158 (SMDV) pooled across 10 animals. **(L)** Frequency (upper) and mean amplitude (lower) of DB02 (n =10), SMDD (n =16) and SMDV (n = 14) GCaMP activity peaks in forward and reversal command states across whole-brain and whole-nervous-system recordings. **p<0.01, Wilcoxon matched-pairs signed rank test. ns, not significant.

Figure S6. Tyramine mutations only partially affect upper-hierarchy SMD activity switch, related to Figure 6. (A) Experimental design. The RIM interneuron synapses onto both SMDD and SMDV. Several studies have shown that RIM signals via tyramine to affect head oscillations and reversal behavior (Alkema et al., 2005; Donnelly et al., 2013; Pirri et al., 2009). We therefore tested a deletion mutant of *tdc-1*, a tyrosine decarboxylase required for tyramine biosynthesis. These *tdc-1 (n3419)* mutants are strongly defective in tyramine production (Alkema et al., 2005). We also tested a large deletion allele of *lgc-55* (Donnelly et al., 2013; Ringstad et al., 2009), a tyramine receptor that is expressed in the SMD neurons (Pirri et al., 2009) (Figure S6A). Both mutant strains were defective in the forward/reverse state modulation of SMDD frequency, but no other parameters (Figure S6B). **(B)** Frequency (upper) and average amplitude (lower) of activity peaks in *tdc-1 (n3419)* (n=16), *lgc-55 (n4331)* (n=16), and parallel wild-type controls (n=17). ns, not significant; *p<0.05, **p<0.01,

***p<0.001, ****p<0.0001, Wilcoxon matched-pairs signed rank test. Each data point is from one animal.

Figure S7. Neuronal activity during different head-bend types, related to Figure 7. (A-B) Trigger-averaged GCaMP/mCherry traces and kymograms from DB02 (**A**) and SMD (**B**) neuronal activity recordings in freely moving animals during either shallow (left, up to angle #4) vs. deep (right, angle #5 and greater) head-casts following dorsal propagated-bends. n = 206 (DB02, shallow), 111 (DB02, deep), 161 (SMD, shallow) and 15 (SMD, deep). Data pooled from 11 animals for SMD and 10 animals for DB02. **(C-D)** Example SMD traces (upper) and kymograms (lower) with two dorsal (**C**) or ventral (**D**) head-cast pairs. **(E-F)** In freely moving (**E**) and immobilized (**F**) animals, probability of at least one unilateral SMD oscillation between a pair of SMD alternations, binned according to the SMD alternation pair's time interval. Cumulative fraction of all SMD alternation pair time intervals shown in orange. Compare to **Fig. S1H**. n = 351 oscillations and 1191 alternation pairs (**E**) and 92 oscillations 324 alternation pairs (**F**) pairs pooled across 11 (**E**) and 13 (**F**) animals. **(G-I)** Fractional distributions of SMD Ca^{2+} peaks binned by head-bend phase during forward locomotion in RIA::tetx animals (**G**, lower) versus parallel wild-type controls (**G**, upper), RIA::hisCl animals with (**H**, lower) or without (**H**, upper) histamine, and *unc-25 (e156)* animals (**I**, lower) versus parallel wild-type controls (**I**, upper). We confirmed that RIA::hisCl animals showed potently RIA inhibition upon histamine provision (data not shown). $p<10^{-6}$ for all distributions, indicating the probability that distributions are drawn randomly (Methods). n = 226 (SMDD) and 201 (SMDV) (**G**, upper), 209 (SMDD) and 261 (SMDV) (**G**, lower), 323 (SMDD) and 288 (SMDV) (**H**, upper), 296 (SMDD) and 273 (SMDV) (**H**, lower), 308 (SMDD) and

272 (SMDV) (**I**, upper), 228 (SMDD) and 215 (SMDV) (**I**, lower). Data are pooled from n = 4 (**G**, upper) or 5 (**G**, lower, and **H-I**) animals.

Figure S8. Sensory input and internal state effects on head-bend type, related to Figure 8. (A) Fractional heat-map of locomotion speed versus propagated-bend inter-bend interval, averaged across 14 experimental repeats with ~20 animals each. Pearson correlation coefficient and p-value indicated. Correlation analysis between speed and inter-bend interval from the underlying unbinned data. **(B)** Population mean ± SD of locomotion speed (upper), propagated-bend frequency (lower, black) and head-cast frequency (lower, green) upon 21% to 4% O_2 shift. n =14 experimental repeats with ~20 animals each. The population mean is first calculated across all animals detected in each time point within an assay, then averaged across assays. **(C-D)** Fractional heat-map showing the distribution of all data (**C**) or all head-casts (**D**) as a function of locomotion speed and angular speed from behavioral assays on food, averaged across n = 19 experimental repeats with ~20 animals each. Each data point is averaged over a 10s sliding window. Data above upper white line are considered dwelling and data within lower right box are considered roaming. Note that roaming is devoid of head casting events. **(E)** Two different hierarchy models. The hierarchy we describe in **Fig. 8A** is non-overlapping, like the hierarchy on the left: no lower-level state is connected to more than a single hierarch. In an overlapping hierarchy, like the one on the right, a lower-level state (labeled in red) is connected to two different hierarchs (Dawkins, 1976). Only in the overlapping hierarchy can a single lower-level behavioral state be accessed during different upper-level states.

Supplemental tables

Table S1. Detailed strain list, related to Figures 1-8, S1-S8, with specific figure panels listed.

Strain name	*Genotype*	*Construct (plasmid no.) injection concentrations*	*Additional References*	Figure panel
ZIM958	*lite-1 (ce314)*	-	*(Bhatla and Horvitz, 2015)*	1, 8I-N, S1, S8
ZIM1466	*lite-1 (ce314); mzmEx877; mzmIs52*	*Pnlr-1(-150;-1)::HisCl::SL2::mCherry (pAN30) – linearized, 0.5ng/uL*<br>*Punc-122::dsred – linearized, 4ng/uL*<br><br>*Punc-31::NLSGCaMP6f (pTS100, codon-optimized and with introns) – linearized, 2.5ng/uL*	*Pnlr-1 (Gendrel et al., 2016; Haklai-Topper et al., 2011) ; a gift from Drs. Marie Gendrel and Oliver Hobert, 150bp upstream of nlr-1 ATG.*	2, 3, 4A, 5K, 7H, S2, S3, S5K-L, S7F
ZIM1564	*lite-1 (ce314); mzmEx929*	*Punc-7s::CreVDH (pHK248) - 100ng/uL*<br>*Pmyo-3::mCherry - 2ng/uL*	*Punc-7s (Starich et al., 2009)*<br><br>*CreVDH (Ruijtenberg and van den Heuvel, 2015)*	4B, 5I-J, S4
ZIM1725	*lite-1 (ce314); mzmEx1018*	*Punc-7s::CreVDH (pHK248) - 40ng/uL*<br>*Pmyo-3::mCherry - 2ng/uL*	*Punc-7s (Starich et al., 2009)*<br><br>*CreVDH (Ruijtenberg and van den Heuvel, 2015)*	4B, 5I-J, S4, S5G-J
ZIM1418	*lite-1 (ce314); mzmEx858*	*Pflp-22::DIO-HisCl::SL2::mCherry (pHK244) - 60 ng/uL*<br>*Pelt-2::NLSdsRedNLS - 5 ng/uL*	*Pflp-22 (Kim and Li, 2004) ; a gift from Dr. Kyuhyung Kim*<br><br>*DIO (Sohal et al., 2009)*	4B, 5I-J, S4, S5G-J
ZIM1473	*lite-1 (ce314); mzmIs28*	*Punc-17beta::HisCl::SL2::mCherry (pHK172) - 80ng/uL*	*Punc-17beta (Charlie et al., 2006) ; a gift from Dr. Kenneth Miller*	4B, S4
ZIM1628	*lite-1 (ce314); mzmEx877; mzmEx929; mzmIs52*	*Pnlr-1(-150;-1)::HisCl::SL2::mCherry (pAN30) – linearized, 0.5ng/uL*<br>*Punc-122::dsred – linearized, 4ng/uL*<br><br>*Punc-7s::CreVDH (pHK248) - 100ng/uL*<br>*Pmyo-3::mCherry - 2ng/uL*<br><br>*Punc-31::NLSGCaMP6f (pTS100, codon-optimized and with introns) – linearized, 2.5ng/uL*	*Pnlr-1 (Gendrel et al., 2016; Haklai-Topper et al., 2011) ; a gift from Drs. Marie Gendrel and Oliver Hobert*<br><br>*Punc-7s (Starich et al., 2009)*<br><br>*CreVDH (Ruijtenberg and van den Heuvel, 2015)*	4A, 5K, S3C-F
ZIM1748	*lite-1 (ce314); mzmEx877; mzmEx1018; mzmIs52*	*Pnlr-1(-150;-1)::HisCl::SL2::mCherry (pAN30) – linearized, 0.5ng/uL*<br>*Punc-122::dsred – linearized, 4ng/uL*<br><br>*Punc-7s::CreVDH (pHK248) - 40ng/uL*<br>*Pmyo-3::mCherry - 2ng/uL*<br><br>*Punc-31::NLSGCaMP6f (pTS100, codon-optimized and with introns) – linearized, 2.5ng/uL*	*Pnlr-1 (Gendrel et al., 2016; Haklai-Topper et al., 2011) ; a gift from Drs. Marie Gendrel and Oliver Hobert*<br><br>*Punc-7s (Starich et al., 2009)*<br><br>*CreVDH (Ruijtenberg and van den Heuvel, 2015)*	4A, 5K, S3C-H
ZIM1562	*lite-1 (ce314); mzmEx877; mzmEx858; mzmIs52*	*Pnlr-1(-150;-1)::HisCl::SL2::mCherry (pAN30) – linearized, 0.5ng/uL*<br>*Punc-122::dsred – linearized, 4ng/uL*<br><br>*Pflp-22::DIO-HisCl::SL2::mCherry (pHK244) - 60 ng/uL*<br>*Pelt-2::NLSdsRedNLS - 5 ng/uL*<br><br>*Punc-31::NLSGCaMP6f (pTS100, codon-optimized and with introns) – linearized, 2.5ng/uL*	*Pnlr-1 (Gendrel et al., 2016; Haklai-Topper et al., 2011) ; a gift from Drs. Marie Gendrel and Oliver Hobert*<br><br>*Pflp-22 (Kim and Li, 2004) ; a gift from Dr. Kyuhyung Kim*<br><br>*DIO (Sohal et al., 2009)*	4A, 5K, S3C-H
ZIM1574	*lite-1 (ce314); mzmEx877; mzmIs28; mzmIs52*	*Pnlr-1(-150;-1)::HisCl::SL2::mCherry (pAN30) – linearized, 0.5ng/uL*<br>*Punc-122::dsred – linearized, 4ng/uL*	*Pnlr-1 (Gendrel et al., 2016; Haklai-Topper et al., 2011) ; a gift from*	4A

		Punc-17beta::HisCl::SL2::mCherry (pHK172) - 80ng/uL<br><br>*Punc-31::NLSGCaMP6f (pTS100, codon-optimized and with introns) – linearized, 2.5ng/uL*	*Drs. Marie Gendrel and Oliver Hobert*<br><br>*Punc-17beta (Charlie et al., 2006) ; a gift from Dr. Kenneth Miller*	
ZIM1658	*lite-1 (ce314); mzmEx981*	*Punc-17beta::NLSGCaMP6f (pHK264, codon-optimized and with introns) – 20ng/uL*<br><br>*Punc-17beta::mCherry::his58 (pHK114) – 40ng/uL*	*Punc-17beta (Charlie et al., 2006) ; a gift from Dr. Kenneth Miller*	5, 7B,D, S5C-D S7A
ZIM1467	*lite-1 (ce314); mzmEx882*	*Punc-7S::CreVDH (pHK248) - 100ng/uL*<br>*Pflp-22::DIO-mCherry (pHK246) - 50ng/uL*<br>*Pflp-22::DIO-GCaMP6Fopt (pHK247, codon-optimized and with introns) - 30ng/uL*	*CreVDH (Ruijtenberg and van den Heuvel, 2015)*<br><br>*Pflp-22 (Kim and Li, 2004) ; a gift from Dr. Kyuhyung Kim*<br><br>*CreVDH (Ruijtenberg and van den Heuvel, 2015)*<br><br>*DIO (Sohal et al., 2009)*	5, 7, S5A-B, S5D-F, S6B, S7B-E, S7G, I
ZIM2122	*lite-1 (ce314); mzmEx1268; mzmEx882*	*Pinx-1::hisCl (pHK307) - 50ng/uL*<br>*Pflp-17::mCherry (MZ39) - 1.5ng/uL*<br><br>*Punc-7S::CreVDH (pHK248) - 100ng/uL*<br>*Pflp-22::DIO-mCherry (pHK246) - 50ng/uL*<br>*Pflp-22::DIO-GCaMP6Fopt (pHK247, codon-optimized and with introns) - 30ng/uL*	*CreVDH (Ruijtenberg and van den Heuvel, 2015)*<br><br>*Pflp-22 (Kim and Li, 2004) ; a gift from Dr. Kyuhyung Kim*<br><br>*DIO (Sohal et al., 2009)*	6B
ZIM2105	*lite-1 (ce314); tdc-1 (n3419); mzmEx882*	*Punc-7S::CreVDH (pHK248) - 100ng/uL*<br>*Pflp-22::DIO-mCherry (pHK246) - 50ng/uL*<br>*Pflp-22::DIO-GCaMP6Fopt (pHK247) - 30ng/uL*	*CreVDH (Ruijtenberg and van den Heuvel, 2015)*<br>*Pflp-22 (Kim and Li, 2004) ; a gift from Dr. Kyuhyung Kim*<br><br>*DIO (Sohal et al., 2009)*<br><br>*tdc-1 (Alkema et al., 2005)*	S6B
ZIM2106	*lite-1 (ce314); lgc-55 (n4331); mzmEx882*	*Punc-7S::CreVDH (pHK248) - 100ng/uL*<br>*Pflp-22::DIO-mCherry (pHK246) - 50ng/uL*<br>*Pflp-22::DIO-GCaMP6Fopt (pHK247) - 30ng/uL*	*CreVDH (Ruijtenberg and van den Heuvel, 2015)*<br><br>*Pflp-22 (Kim and Li, 2004) ; a gift from Dr. Kyuhyung Kim*<br><br>*DIO (Sohal et al., 2009)*<br><br>*lgc-55 (Ringstad et al., 2009)*	S6B
ZIM2124	*lite-1 (ce314); yxEx696; mzmEx882*	*Pglr-3::TeTx::mCherry (yxEx696) - 50 ng/uL*<br>*Punc-122::dsred – linearized, 4ng/uL*<br><br>*Punc-7S::CreVDH (pHK248) - 100ng/uL*<br>*Pflp-22::DIO-mCherry (pHK246) - 50ng/uL*<br>*Pflp-22::DIO-GCaMP6Fopt (pHK247) - 30ng/uL*	*CreVDH (Ruijtenberg and van den Heuvel, 2015)*<br><br>*Pflp-22 (Kim and Li, 2004) ; a gift from Dr. Kyuhyung Kim*<br><br>*DIO (Sohal et al., 2009)*<br><br>*glr-3::TeTx (Liu et al., 2018)*	S7G
ZIM2120	*lite-1 (ce314); mzmEx1262; mzmEx882*	*Pglr-3::hisCl::SL2::mCherry (pRL120) - 25ng/uL*<br>*Pflp-17::mCherry (MZ39) - 1.5ng/uL*<br><br>*Punc-7S::CreVDH (pHK248) - 100ng/uL*	*CreVDH (Ruijtenberg and van den Heuvel, 2015)*	S7H

		Pflp-22::DIO-mCherry (pHK246) - 50ng/uL<br>*Pflp-22::DIO-GCaMP6Fopt (pHK247) - 30ng/uL*	*Pflp-22 (Kim and Li, 2004) ; a gift from Dr. Kyuhyung Kim*<br><br>*DIO (Sohal et al., 2009)*	
ZIM2128	*lite-1 (ce314) ; unc-25(e156); mzmEx882*	*Punc-7S::CreVDH (pHK248) - 100ng/uL*<br>*Pflp-22::DIO-mCherry (pHK246) - 50ng/uL*<br>*Pflp-22::DIO-GCaMP6Fopt (pHK247) - 30ng/uL*	*CreVDH (Ruijtenberg and van den Heuvel, 2015)*<br><br>*Pflp-22 (Kim and Li, 2004) ; a gift from Dr. Kyuhyung Kim*<br><br>*DIO (Sohal et al., 2009)*<br><br>*unc-25 (e156) (Brenner)*	S7I
ZIM1749	*lite-1 (ce314); mzmEx1041*	*Psto-3::HisCl::mCherry (pHK170) - 10ng/uL*<br>*Punc-122::GFP - 15ng/uL*	*Psto-3 (Kato et al., 2015)*	8D-E
ZIM1563	*lite-1 (ce314); mzmEx928*	*Psto-3::Chrimson::mCherry (pHK256) - 20ng/uL*<br>*Punc-122::GFP - 20ng/uL*	*Psto-3 (Kato et al., 2015)*	8G-H

Table S2. Related to Figures 3 and S2. Second column: Number of instances where each neuron was identified in immobilized Ca^{2+} imaging recordings for the calculation of mean activity level differences in forward vs. reverse command states (**Fig. S2C**). Third column: p-values for the paired t-test (**Fig. S2C**). Fourth column: number of forward states per neuron for which peak frequencies were calculated (**Fig. 3A**). Fifth column: p-values for inter-peak interval distribution resampling test (**Fig. 3A**). This was calculated only for neurons with multiple peaks per forward state, and statistical significance is corrected for multiple comparisons (Methods).

Neuron name	# observations	P-value (Fig. S2C)	# forward states	P-value (Fig. 3A)
AIBL	10	0.000119	56	-
AIBR	10	0.000056	56	-
ALA	10	0.041445	56	0.1691
ASKL	8	0.007442	45	0.000686
ASKR	5	0.053196	25	0.1084
AVAL	10	0.000007	56	-
AVAR	10	0.000021	56	-
AVBL	8	0.003679	42	0.0923
AVBR	5	0.004497	30	0.0862
AVEL	10	0.000001	56	-
AVER	9	0.000016	49	-
AVFL	7	0.077079	37	-
AVFR	8	0.293196	46	-
BAGL	7	0.700558	37	-
BAGR	7	0.831927	37	-
Baseline	-	-	56	-

CEPDL	3	0.153182	20	-
CEPDR	3	0.730871	18	$<10^{-6}$
DB01	9	0.444463	49	$1x10^{-6}$
DB02	10	0.001824	56	$5x10^{-6}$
DB03	5	0.008244	26	0.0078
DB04	4	0.111655	21	0.3152
DB05	4	0.247590	21	0.0731
DB06	4	0.053807	22	0.1825
DB07a	5	0.000419	26	$1x10^{-6}$
DVA	5	0.047722	26	0.0301
OLQDR	3	0.209337	18	-
OLQVL	3	0.249957	19	-
OLQVR	4	0.014049	25	-
PDA	3	0.303379	14	$<10^{-6}$
RIBL	9	0.000788	51	$1.1x10^{-5}$
RIBR	9	0.001071	51	$9x10^{-6}$
RID	4	0.007461	24	-
RIML	10	0.000113	56	-
RIMR	9	0.000187	50	-
RIVL	9	0.041139	49	0.0106
RIVR	9	0.002213	49	0.0407
RMDVR	3	0.465273	19	-
RMED	10	0.000043	56	$<10^{-6}$
RMEL	3	0.000169	16	0.0021
RMER	4	0.009923	23	0.0015
RMEV	8	0.045459	46	0.000377
SIBVL	5	0.229044	30	0.2435
SIBVR	4	0.034030	24	0.1493
SMDDL	10	0.007962	56	$<10^{-6}$
SMDDR	9	0.010916	49	$<10^{-6}$
SMDVL	10	0.001672	56	$2x10^{-6}$
SMDVR	10	0.001490	56	.000234
URYDL	10	0.080583	56	-
URYDR	8	0.107907	45	-
URYVL	9	0.122731	49	-
URYVR	8	0.003887	45	-
VA02	6	0.055074	31	-
VB01	10	0.012811	56	0.000163

VB02	10	0.000033	56	0.1635
VB03	6	0.120118	31	0.0137
VB04	4	0.313493	19	0.4979
VB05	5	0.004762	26	0.0304
VB06	5	0.005444	26	0.0495
VB07	4	0.001905	21	0.0189
VB08	5	0.002505	26	0.0667
VB09	5	0.003075	26	0.0034
VB10	4	0.007944	23	0.0366
VB11p	5	0.007691	26	0.000412
VD02	3	0.464618	18	0.5031

Table S3. Related to Figures 3 and S2. P-values for covariogram significance test for covariograms shown in **Fig. 3C and Fig. S2E**. Statistical significance is corrected for multiple comparisons (Methods).

	**SMDDL**	**SMDDR**	**SMDVL**	**SMDVR**	**DB01**	**DB02**	**RMED**	**RMEV**	**VB01**
**SMDDL**	$<10^{-6}$	$<10^{-6}$	$1x10^{-6}$	$1x10^{-6}$	0.3393	0.4995	$<10^{-6}$	0.003	$1x10^{-6}$
**SMDDR**	$<10^{-6}$	$<10^{-6}$	$1x10^{-6}$	$4x10^{-6}$	0.2137	0.6218	$<10^{-6}$	0.157	$3x10^{-5}$
**SMDVL**	$8x10^{-6}$	$1x10^{-5}$	$<10^{-6}$	$<10^{-6}$	0.489	0.005	0.0012	$5x10^{-6}$	$<10^{-6}$
**SMDVR**	$<10^{-6}$	$5x10^{-6}$	$<10^{-6}$	$<10^{-6}$	0.0838	$2x10^{-5}$	0.0545	$<10^{-6}$	$<10^{-6}$
**DB01**	0.6151	0.1462	0.4047	0.0793	$<10^{-6}$	$<10^{-6}$	0.4562	0.0152	0.6005
**DB02**	0.7179	0.8183	0.0042	$1x10^{-5}$	$<10^{-6}$	$<10^{-6}$	0.1144	0.1501	0.0003
	**DB01**	**DB02**	**DB07a**	**VB01**	**VB09**	**VB11p**			
**DB01**	$<10^{-6}$	$<10^{-6}$	0.2229	0.6001	0.5037	0.4931			
**DB02**	$<10^{-6}$	$<10^{-6}$	0.9201	0.0003	0.1873	0.8323			
**DB07a**	0.6201	0.7833	$<10^{-6}$	0.2583	0.1385	0.4710			
**VB01**	0.3821	0.0001	0.6632	$<10^{-6}$	0.633	0.0137			
**VB09**	0.4753	0.1413	0.7336	0.72	$<10^{-6}$	0.2397			
**VB11p**	0.2352	0.6546	0.3671	0.0068	0.142	$<10^{-6}$			
